# Study of $WW\gamma$ and $WZ\gamma$ production in $pp$ collisions at $\sqrt{s} = {8} \,{\text {TeV}}$ and search for anomalous quartic gauge couplings with the ATLAS experiment

**DOI:** 10.1140/epjc/s10052-017-5180-3

**Published:** 2017-09-25

**Authors:** M. Aaboud, G. Aad, B. Abbott, O. Abdinov, B. Abeloos, S. H. Abidi, O. S. AbouZeid, N. L. Abraham, H. Abramowicz, H. Abreu, R. Abreu, Y. Abulaiti, B. S. Acharya, S. Adachi, L. Adamczyk, J. Adelman, M. Adersberger, T. Adye, A. A. Affolder, T. Agatonovic-Jovin, C. Agheorghiesei, J. A. Aguilar-Saavedra, S. P. Ahlen, F. Ahmadov, G. Aielli, S. Akatsuka, H. Akerstedt, T. P. A. Åkesson, E. Akilli, A. V. Akimov, G. L. Alberghi, J. Albert, P. Albicocco, M. J. Alconada Verzini, S. C. Alderweireldt, M. Aleksa, I. N. Aleksandrov, C. Alexa, G. Alexander, T. Alexopoulos, M. Alhroob, B. Ali, M. Aliev, G. Alimonti, J. Alison, S. P. Alkire, B. M. M. Allbrooke, B. W. Allen, P. P. Allport, A. Aloisio, A. Alonso, F. Alonso, C. Alpigiani, A. A. Alshehri, M. I. Alstaty, B. Alvarez Gonzalez, D. Álvarez Piqueras, M. G. Alviggi, B. T. Amadio, Y. Amaral Coutinho, C. Amelung, D. Amidei, S. P. Amor Dos Santos, S. Amoroso, G. Amundsen, C. Anastopoulos, L. S. Ancu, N. Andari, T. Andeen, C. F. Anders, J. K. Anders, K. J. Anderson, A. Andreazza, V. Andrei, S. Angelidakis, I. Angelozzi, A. Angerami, A. V. Anisenkov, N. Anjos, A. Annovi, C. Antel, M. Antonelli, A. Antonov, D. J. Antrim, F. Anulli, M. Aoki, L. Aperio Bella, G. Arabidze, Y. Arai, J. P. Araque, V. Araujo Ferraz, A. T. H. Arce, R. E. Ardell, F. A. Arduh, J-F. Arguin, S. Argyropoulos, M. Arik, A. J. Armbruster, L. J. Armitage, O. Arnaez, H. Arnold, M. Arratia, O. Arslan, A. Artamonov, G. Artoni, S. Artz, S. Asai, N. Asbah, A. Ashkenazi, L. Asquith, K. Assamagan, R. Astalos, M. Atkinson, N. B. Atlay, K. Augsten, G. Avolio, B. Axen, M. K. Ayoub, G. Azuelos, A. E. Baas, M. J. Baca, H. Bachacou, K. Bachas, M. Backes, M. Backhaus, P. Bagnaia, M. Bahmani, H. Bahrasemani, J. T. Baines, M. Bajic, O. K. Baker, E. M. Baldin, P. Balek, F. Balli, W. K. Balunas, E. Banas, A. Bandyopadhyay, Sw. Banerjee, A. A. E. Bannoura, L. Barak, E. L. Barberio, D. Barberis, M. Barbero, T. Barillari, M-S. Barisits, J. T. Barkeloo, T. Barklow, N. Barlow, S. L. Barnes, B. M. Barnett, R. M. Barnett, Z. Barnovska-Blenessy, A. Baroncelli, G. Barone, A. J. Barr, L. Barranco Navarro, F. Barreiro, J. Barreiro Guimarães da Costa, R. Bartoldus, A. E. Barton, P. Bartos, A. Basalaev, A. Bassalat, R. L. Bates, S. J. Batista, J. R. Batley, M. Battaglia, M. Bauce, F. Bauer, H. S. Bawa, J. B. Beacham, M. D. Beattie, T. Beau, P. H. Beauchemin, P. Bechtle, H. P. Beck, H. C. Beck, K. Becker, M. Becker, M. Beckingham, C. Becot, A. J. Beddall, A. Beddall, V. A. Bednyakov, M. Bedognetti, C. P. Bee, T. A. Beermann, M. Begalli, M. Begel, J. K. Behr, A. S. Bell, G. Bella, L. Bellagamba, A. Bellerive, M. Bellomo, K. Belotskiy, O. Beltramello, N. L. Belyaev, O. Benary, D. Benchekroun, M. Bender, K. Bendtz, N. Benekos, Y. Benhammou, E. Benhar Noccioli, J. Benitez, D. P. Benjamin, M. Benoit, J. R. Bensinger, S. Bentvelsen, L. Beresford, M. Beretta, D. Berge, E. Bergeaas Kuutmann, N. Berger, J. Beringer, S. Berlendis, N. R. Bernard, G. Bernardi, C. Bernius, F. U. Bernlochner, T. Berry, P. Berta, C. Bertella, G. Bertoli, F. Bertolucci, I. A. Bertram, C. Bertsche, D. Bertsche, G. J. Besjes, O. Bessidskaia Bylund, M. Bessner, N. Besson, C. Betancourt, A. Bethani, S. Bethke, A. J. Bevan, J. Beyer, R. M. Bianchi, O. Biebel, D. Biedermann, R. Bielski, K. Bierwagen, N. V. Biesuz, M. Biglietti, T. R. V. Billoud, H. Bilokon, M. Bindi, A. Bingul, C. Bini, S. Biondi, T. Bisanz, C. Bittrich, D. M. Bjergaard, C. W. Black, J. E. Black, K. M. Black, R. E. Blair, T. Blazek, I. Bloch, C. Blocker, A. Blue, W. Blum, U. Blumenschein, S. Blunier, G. J. Bobbink, V. S. Bobrovnikov, S. S. Bocchetta, A. Bocci, C. Bock, M. Boehler, D. Boerner, D. Bogavac, A. G. Bogdanchikov, C. Bohm, V. Boisvert, P. Bokan, T. Bold, A. S. Boldyrev, A. E. Bolz, M. Bomben, M. Bona, M. Boonekamp, A. Borisov, G. Borissov, J. Bortfeldt, D. Bortoletto, V. Bortolotto, D. Boscherini, M. Bosman, J. D. Bossio Sola, J. Boudreau, J. Bouffard, E. V. Bouhova-Thacker, D. Boumediene, C. Bourdarios, S. K. Boutle, A. Boveia, J. Boyd, I. R. Boyko, J. Bracinik, A. Brandt, G. Brandt, O. Brandt, U. Bratzler, B. Brau, J. E. Brau, W. D. Breaden Madden, K. Brendlinger, A. J. Brennan, L. Brenner, R. Brenner, S. Bressler, D. L. Briglin, T. M. Bristow, D. Britton, D. Britzger, F. M. Brochu, I. Brock, R. Brock, G. Brooijmans, T. Brooks, W. K. Brooks, J. Brosamer, E. Brost, J. H Broughton, P. A. Bruckman de Renstrom, D. Bruncko, A. Bruni, G. Bruni, L. S. Bruni, BH Brunt, M. Bruschi, N. Bruscino, P. Bryant, L. Bryngemark, T. Buanes, Q. Buat, P. Buchholz, A. G. Buckley, I. A. Budagov, F. Buehrer, M. K. Bugge, O. Bulekov, D. Bullock, T. J. Burch, S. Burdin, C. D. Burgard, A. M. Burger, B. Burghgrave, K. Burka, S. Burke, I. Burmeister, J. T. P. Burr, E. Busato, D. Büscher, V. Büscher, P. Bussey, J. M. Butler, C. M. Buttar, J. M. Butterworth, P. Butti, W. Buttinger, A. Buzatu, A. R. Buzykaev, S. Cabrera Urbán, D. Caforio, V. M. Cairo, O. Cakir, N. Calace, P. Calafiura, A. Calandri, G. Calderini, P. Calfayan, G. Callea, L. P. Caloba, S. Calvente Lopez, D. Calvet, S. Calvet, T. P. Calvet, R. Camacho Toro, S. Camarda, P. Camarri, D. Cameron, R. Caminal Armadans, C. Camincher, S. Campana, M. Campanelli, A. Camplani, A. Campoverde, V. Canale, M. Cano Bret, J. Cantero, T. Cao, M. D. M. Capeans Garrido, I. Caprini, M. Caprini, M. Capua, R. M. Carbone, R. Cardarelli, F. Cardillo, I. Carli, T. Carli, G. Carlino, B. T. Carlson, L. Carminati, R. M. D. Carney, S. Caron, E. Carquin, S. Carrá, G. D. Carrillo-Montoya, D. Casadei, M. P. Casado, M. Casolino, D. W. Casper, R. Castelijn, V. Castillo Gimenez, N. F. Castro, A. Catinaccio, J. R. Catmore, A. Cattai, J. Caudron, V. Cavaliere, E. Cavallaro, D. Cavalli, M. Cavalli-Sforza, V. Cavasinni, E. Celebi, F. Ceradini, L. Cerda Alberich, A. S. Cerqueira, A. Cerri, L. Cerrito, F. Cerutti, A. Cervelli, S. A. Cetin, A. Chafaq, D. Chakraborty, S. K. Chan, W. S. Chan, Y. L. Chan, P. Chang, J. D. Chapman, D. G. Charlton, C. C. Chau, C. A. Chavez Barajas, S. Che, S. Cheatham, A. Chegwidden, S. Chekanov, S. V. Chekulaev, G. A. Chelkov, M. A. Chelstowska, C. Chen, H. Chen, J. Chen, S. Chen, S. Chen, X. Chen, Y. Chen, H. C. Cheng, H. J. Cheng, A. Cheplakov, E. Cheremushkina, R. Cherkaoui El Moursli, E. Cheu, K. Cheung, L. Chevalier, V. Chiarella, G. Chiarelli, G. Chiodini, A. S. Chisholm, A. Chitan, Y. H. Chiu, M. V. Chizhov, K. Choi, A. R. Chomont, S. Chouridou, Y. S. Chow, V. Christodoulou, M. C. Chu, J. Chudoba, A. J. Chuinard, J. J. Chwastowski, L. Chytka, A. K. Ciftci, D. Cinca, V. Cindro, I. A. Cioara, C. Ciocca, A. Ciocio, F. Cirotto, Z. H. Citron, M. Citterio, M. Ciubancan, A. Clark, B. L. Clark, M. R. Clark, P. J. Clark, R. N. Clarke, C. Clement, Y. Coadou, M. Cobal, A. Coccaro, J. Cochran, L. Colasurdo, B. Cole, A. P. Colijn, J. Collot, T. Colombo, P. Conde Muiño, E. Coniavitis, S. H. Connell, I. A. Connelly, S. Constantinescu, G. Conti, F. Conventi, M. Cooke, A. M. Cooper-Sarkar, F. Cormier, K. J. R. Cormier, M. Corradi, F. Corriveau, A. Cortes-Gonzalez, G. Cortiana, G. Costa, M. J. Costa, D. Costanzo, G. Cottin, G. Cowan, B. E. Cox, K. Cranmer, S. J. Crawley, R. A. Creager, G. Cree, S. Crépé-Renaudin, F. Crescioli, W. A. Cribbs, M. Cristinziani, V. Croft, G. Crosetti, A. Cueto, T. Cuhadar Donszelmann, A. R. Cukierman, J. Cummings, M. Curatolo, J. Cúth, S. Czekierda, P. Czodrowski, G. D’amen, S. D’Auria, L. D’eramo, M. D’Onofrio, M. J. Da Cunha Sargedas De Sousa, C. Da Via, W. Dabrowski, T. Dado, T. Dai, O. Dale, F. Dallaire, C. Dallapiccola, M. Dam, J. R. Dandoy, M. F. Daneri, N. P. Dang, A. C. Daniells, N. S. Dann, M. Danninger, M. Dano Hoffmann, V. Dao, G. Darbo, S. Darmora, J. Dassoulas, A. Dattagupta, T. Daubney, W. Davey, C. David, T. Davidek, D. R. Davis, P. Davison, E. Dawe, I. Dawson, K. De, R. de Asmundis, A. De Benedetti, S. De Castro, S. De Cecco, N. De Groot, P. de Jong, H. De la Torre, F. De Lorenzi, A. De Maria, D. De Pedis, A. De Salvo, U. De Sanctis, A. De Santo, K. De Vasconcelos Corga, J. B. De Vivie De Regie, W. J. Dearnaley, R. Debbe, C. Debenedetti, D. V. Dedovich, N. Dehghanian, I. Deigaard, M. Del Gaudio, J. Del Peso, D. Delgove, F. Deliot, C. M. Delitzsch, A. Dell’Acqua, L. Dell’Asta, M. Dell’Orso, M. Della Pietra, D. della Volpe, M. Delmastro, C. Delporte, P. A. Delsart, D. A. DeMarco, S. Demers, M. Demichev, A. Demilly, S. P. Denisov, D. Denysiuk, D. Derendarz, J. E. Derkaoui, F. Derue, P. Dervan, K. Desch, C. Deterre, K. Dette, M. R. Devesa, P. O. Deviveiros, A. Dewhurst, S. Dhaliwal, F. A. Di Bello, A. Di Ciaccio, L. Di Ciaccio, W. K. Di Clemente, C. Di Donato, A. Di Girolamo, B. Di Girolamo, B. Di Micco, R. Di Nardo, K. F. Di Petrillo, A. Di Simone, R. Di Sipio, D. Di Valentino, C. Diaconu, M. Diamond, F. A. Dias, M. A. Diaz, E. B. Diehl, J. Dietrich, S. Díez Cornell, A. Dimitrievska, J. Dingfelder, P. Dita, S. Dita, F. Dittus, F. Djama, T. Djobava, J. I. Djuvsland, M. A. B. do Vale, D. Dobos, M. Dobre, C. Doglioni, J. Dolejsi, Z. Dolezal, M. Donadelli, S. Donati, P. Dondero, J. Donini, J. Dopke, A. Doria, M. T. Dova, A. T. Doyle, E. Drechsler, M. Dris, Y. Du, J. Duarte-Campderros, A. Dubreuil, E. Duchovni, G. Duckeck, A. Ducourthial, O. A. Ducu, D. Duda, A. Dudarev, A. Chr. Dudder, E. M. Duffield, L. Duflot, M. Dührssen, M. Dumancic, A. E. Dumitriu, A. K. Duncan, M. Dunford, H. Duran Yildiz, M. Düren, A. Durglishvili, D. Duschinger, B. Dutta, M. Dyndal, B. S. Dziedzic, C. Eckardt, K. M. Ecker, R. C. Edgar, T. Eifert, G. Eigen, K. Einsweiler, T. Ekelof, M. El Kacimi, R. El Kosseifi, V. Ellajosyula, M. Ellert, S. Elles, F. Ellinghaus, A. A. Elliot, N. Ellis, J. Elmsheuser, M. Elsing, D. Emeliyanov, Y. Enari, O. C. Endner, J. S. Ennis, J. Erdmann, A. Ereditato, M. Ernst, S. Errede, M. Escalier, C. Escobar, B. Esposito, O. Estrada Pastor, A. I. Etienvre, E. Etzion, H. Evans, A. Ezhilov, M. Ezzi, F. Fabbri, L. Fabbri, V. Fabiani, G. Facini, R. M. Fakhrutdinov, S. Falciano, R. J. Falla, J. Faltova, Y. Fang, M. Fanti, A. Farbin, A. Farilla, C. Farina, E. M. Farina, T. Farooque, S. Farrell, S. M. Farrington, P. Farthouat, F. Fassi, P. Fassnacht, D. Fassouliotis, M. Faucci Giannelli, A. Favareto, W. J. Fawcett, L. Fayard, O. L. Fedin, W. Fedorko, S. Feigl, L. Feligioni, C. Feng, E. J. Feng, H. Feng, M. J. Fenton, A. B. Fenyuk, L. Feremenga, P. Fernandez Martinez, S. Fernandez Perez, J. Ferrando, A. Ferrari, P. Ferrari, R. Ferrari, D. E. Ferreira de Lima, A. Ferrer, D. Ferrere, C. Ferretti, F. Fiedler, A. Filipčič, M. Filipuzzi, F. Filthaut, M. Fincke-Keeler, K. D. Finelli, M. C. N. Fiolhais, L. Fiorini, A. Fischer, C. Fischer, J. Fischer, W. C. Fisher, N. Flaschel, I. Fleck, P. Fleischmann, R. R. M. Fletcher, T. Flick, B. M. Flierl, L. R. Flores Castillo, M. J. Flowerdew, G. T. Forcolin, A. Formica, F. A. Förster, A. Forti, A. G. Foster, D. Fournier, H. Fox, S. Fracchia, P. Francavilla, M. Franchini, S. Franchino, D. Francis, L. Franconi, M. Franklin, M. Frate, M. Fraternali, D. Freeborn, S. M. Fressard-Batraneanu, B. Freund, D. Froidevaux, J. A. Frost, C. Fukunaga, T. Fusayasu, J. Fuster, C. Gabaldon, O. Gabizon, A. Gabrielli, A. Gabrielli, G. P. Gach, S. Gadatsch, S. Gadomski, G. Gagliardi, L. G. Gagnon, C. Galea, B. Galhardo, E. J. Gallas, B. J. Gallop, P. Gallus, G. Galster, K. K. Gan, S. Ganguly, Y. Gao, Y. S. Gao, F. M. Garay Walls, C. García, J. E. García Navarro, J. A. García Pascual, M. Garcia-Sciveres, R. W. Gardner, N. Garelli, V. Garonne, A. Gascon Bravo, K. Gasnikova, C. Gatti, A. Gaudiello, G. Gaudio, I. L. Gavrilenko, C. Gay, G. Gaycken, E. N. Gazis, C. N. P. Gee, J. Geisen, M. Geisen, M. P. Geisler, K. Gellerstedt, C. Gemme, M. H. Genest, C. Geng, S. Gentile, C. Gentsos, S. George, D. Gerbaudo, A. Gershon, G. Geßner, S. Ghasemi, M. Ghneimat, B. Giacobbe, S. Giagu, N. Giangiacomi, P. Giannetti, S. M. Gibson, M. Gignac, M. Gilchriese, D. Gillberg, G. Gilles, D. M. Gingrich, N. Giokaris, M. P. Giordani, F. M. Giorgi, P. F. Giraud, P. Giromini, G. Giugliarelli, D. Giugni, F. Giuli, C. Giuliani, M. Giulini, B. K. Gjelsten, S. Gkaitatzis, I. Gkialas, E. L. Gkougkousis, P. Gkountoumis, L. K. Gladilin, C. Glasman, J. Glatzer, P. C. F. Glaysher, A. Glazov, M. Goblirsch-Kolb, J. Godlewski, S. Goldfarb, T. Golling, D. Golubkov, A. Gomes, R. Gonçalo, R. Goncalves Gama, J. Goncalves Pinto Firmino Da Costa, G. Gonella, L. Gonella, A. Gongadze, S. González de la Hoz, S. Gonzalez-Sevilla, L. Goossens, P. A. Gorbounov, H. A. Gordon, I. Gorelov, B. Gorini, E. Gorini, A. Gorišek, A. T. Goshaw, C. Gössling, M. I. Gostkin, C. A. Gottardo, C. R. Goudet, D. Goujdami, A. G. Goussiou, N. Govender, E. Gozani, L. Graber, I. Grabowska-Bold, P. O. J. Gradin, J. Gramling, E. Gramstad, S. Grancagnolo, V. Gratchev, P. M. Gravila, C. Gray, H. M. Gray, Z. D. Greenwood, C. Grefe, K. Gregersen, I. M. Gregor, P. Grenier, K. Grevtsov, J. Griffiths, A. A. Grillo, K. Grimm, S. Grinstein, Ph. Gris, J.-F. Grivaz, S. Groh, E. Gross, J. Grosse-Knetter, G. C. Grossi, Z. J. Grout, A. Grummer, L. Guan, W. Guan, J. Guenther, F. Guescini, D. Guest, O. Gueta, B. Gui, E. Guido, T. Guillemin, S. Guindon, U. Gul, C. Gumpert, J. Guo, W. Guo, Y. Guo, R. Gupta, S. Gupta, G. Gustavino, P. Gutierrez, N. G. Gutierrez Ortiz, C. Gutschow, C. Guyot, M. P. Guzik, C. Gwenlan, C. B. Gwilliam, A. Haas, C. Haber, H. K. Hadavand, N. Haddad, A. Hadef, S. Hageböck, M. Hagihara, H. Hakobyan, M. Haleem, J. Haley, G. Halladjian, G. D. Hallewell, K. Hamacher, P. Hamal, K. Hamano, A. Hamilton, G. N. Hamity, P. G. Hamnett, L. Han, S. Han, K. Hanagaki, K. Hanawa, M. Hance, B. Haney, P. Hanke, J. B. Hansen, J. D. Hansen, M. C. Hansen, P. H. Hansen, K. Hara, A. S. Hard, T. Harenberg, F. Hariri, S. Harkusha, R. D. Harrington, P. F. Harrison, N. M. Hartmann, M. Hasegawa, Y. Hasegawa, A. Hasib, S. Hassani, S. Haug, R. Hauser, L. Hauswald, L. B. Havener, M. Havranek, C. M. Hawkes, R. J. Hawkings, D. Hayakawa, D. Hayden, C. P. Hays, J. M. Hays, H. S. Hayward, S. J. Haywood, S. J. Head, T. Heck, V. Hedberg, L. Heelan, S. Heer, K. K. Heidegger, S. Heim, T. Heim, B. Heinemann, J. J. Heinrich, L. Heinrich, C. Heinz, J. Hejbal, L. Helary, A. Held, S. Hellman, C. Helsens, R. C. W. Henderson, Y. Heng, S. Henkelmann, A. M. Henriques Correia, S. Henrot-Versille, G. H. Herbert, H. Herde, V. Herget, Y. Hernández Jiménez, H. Herr, G. Herten, R. Hertenberger, L. Hervas, T. C. Herwig, G. G. Hesketh, N. P. Hessey, J. W. Hetherly, S. Higashino, E. Higón-Rodriguez, K. Hildebrand, E. Hill, J. C. Hill, K. H. Hiller, S. J. Hillier, M. Hils, I. Hinchliffe, M. Hirose, D. Hirschbuehl, B. Hiti, O. Hladik, X. Hoad, J. Hobbs, N. Hod, M. C. Hodgkinson, P. Hodgson, A. Hoecker, M. R. Hoeferkamp, F. Hoenig, D. Hohn, T. R. Holmes, M. Homann, S. Honda, T. Honda, T. M. Hong, B. H. Hooberman, W. H. Hopkins, Y. Horii, A. J. Horton, J-Y. Hostachy, S. Hou, A. Hoummada, J. Howarth, J. Hoya, M. Hrabovsky, J. Hrdinka, I. Hristova, J. Hrivnac, T. Hryn’ova, A. Hrynevich, P. J. Hsu, S.-C. Hsu, Q. Hu, S. Hu, Y. Huang, Z. Hubacek, F. Hubaut, F. Huegging, T. B. Huffman, E. W. Hughes, G. Hughes, M. Huhtinen, P. Huo, N. Huseynov, J. Huston, J. Huth, G. Iacobucci, G. Iakovidis, I. Ibragimov, L. Iconomidou-Fayard, Z. Idrissi, P. Iengo, O. Igonkina, T. Iizawa, Y. Ikegami, M. Ikeno, Y. Ilchenko, D. Iliadis, N. Ilic, G. Introzzi, P. Ioannou, M. Iodice, K. Iordanidou, V. Ippolito, M. F. Isacson, N. Ishijima, M. Ishino, M. Ishitsuka, C. Issever, S. Istin, F. Ito, J. M. Iturbe Ponce, R. Iuppa, H. Iwasaki, J. M. Izen, V. Izzo, S. Jabbar, P. Jackson, R. M. Jacobs, V. Jain, K. B. Jakobi, K. Jakobs, S. Jakobsen, T. Jakoubek, D. O. Jamin, D. K. Jana, R. Jansky, J. Janssen, M. Janus, P. A. Janus, G. Jarlskog, N. Javadov, T. Javůrek, M. Javurkova, F. Jeanneau, L. Jeanty, J. Jejelava, A. Jelinskas, P. Jenni, C. Jeske, S. Jézéquel, H. Ji, J. Jia, H. Jiang, Y. Jiang, Z. Jiang, S. Jiggins, J. Jimenez Pena, S. Jin, A. Jinaru, O. Jinnouchi, H. Jivan, P. Johansson, K. A. Johns, C. A. Johnson, W. J. Johnson, K. Jon-And, R. W. L. Jones, S. D. Jones, S. Jones, T. J. Jones, J. Jongmanns, P. M. Jorge, J. Jovicevic, X. Ju, A. Juste Rozas, M. K. Köhler, A. Kaczmarska, M. Kado, H. Kagan, M. Kagan, S. J. Kahn, T. Kaji, E. Kajomovitz, C. W. Kalderon, A. Kaluza, S. Kama, A. Kamenshchikov, N. Kanaya, L. Kanjir, V. A. Kantserov, J. Kanzaki, B. Kaplan, L. S. Kaplan, D. Kar, K. Karakostas, N. Karastathis, M. J. Kareem, E. Karentzos, S. N. Karpov, Z. M. Karpova, K. Karthik, V. Kartvelishvili, A. N. Karyukhin, K. Kasahara, L. Kashif, R. D. Kass, A. Kastanas, Y. Kataoka, C. Kato, A. Katre, J. Katzy, K. Kawade, K. Kawagoe, T. Kawamoto, G. Kawamura, E. F. Kay, V. F. Kazanin, R. Keeler, R. Kehoe, J. S. Keller, E. Kellermann, J. J. Kempster, J Kendrick, H. Keoshkerian, O. Kepka, B. P. Kerševan, S. Kersten, R. A. Keyes, M. Khader, F. Khalil-zada, A. Khanov, A. G. Kharlamov, T. Kharlamova, A. Khodinov, T. J. Khoo, V. Khovanskiy, E. Khramov, J. Khubua, S. Kido, C. R. Kilby, H. Y. Kim, S. H. Kim, Y. K. Kim, N. Kimura, O. M. Kind, B. T. King, D. Kirchmeier, J. Kirk, A. E. Kiryunin, T. Kishimoto, D. Kisielewska, V. Kitali, K. Kiuchi, O. Kivernyk, E. Kladiva, T. Klapdor-Kleingrothaus, M. H. Klein, M. Klein, U. Klein, K. Kleinknecht, P. Klimek, A. Klimentov, R. Klingenberg, T. Klingl, T. Klioutchnikova, E.-E. Kluge, P. Kluit, S. Kluth, E. Kneringer, E. B. F. G. Knoops, A. Knue, A. Kobayashi, D. Kobayashi, T. Kobayashi, M. Kobel, M. Kocian, P. Kodys, T. Koffas, E. Koffeman, N. M. Köhler, T. Koi, M. Kolb, I. Koletsou, A. A. Komar, Y. Komori, T. Kondo, N. Kondrashova, K. Köneke, A. C. König, T. Kono, R. Konoplich, N. Konstantinidis, R. Kopeliansky, S. Koperny, A. K. Kopp, K. Korcyl, K. Kordas, A. Korn, A. A. Korol, I. Korolkov, E. V. Korolkova, O. Kortner, S. Kortner, T. Kosek, V. V. Kostyukhin, A. Kotwal, A. Koulouris, A. Kourkoumeli-Charalampidi, C. Kourkoumelis, E. Kourlitis, V. Kouskoura, A. B. Kowalewska, R. Kowalewski, T. Z. Kowalski, C. Kozakai, W. Kozanecki, A. S. Kozhin, V. A. Kramarenko, G. Kramberger, D. Krasnopevtsev, M. W. Krasny, A. Krasznahorkay, D. Krauss, J. A. Kremer, J. Kretzschmar, K. Kreutzfeldt, P. Krieger, K. Krizka, K. Kroeninger, H. Kroha, J. Kroll, J. Kroll, J. Kroseberg, J. Krstic, U. Kruchonak, H. Krüger, N. Krumnack, M. C. Kruse, T. Kubota, H. Kucuk, S. Kuday, J. T. Kuechler, S. Kuehn, A. Kugel, F. Kuger, T. Kuhl, V. Kukhtin, R. Kukla, Y. Kulchitsky, S. Kuleshov, Y. P. Kulinich, M. Kuna, T. Kunigo, A. Kupco, T. Kupfer, O. Kuprash, H. Kurashige, L. L. Kurchaninov, Y. A. Kurochkin, M. G. Kurth, V. Kus, E. S. Kuwertz, M. Kuze, J. Kvita, T. Kwan, D. Kyriazopoulos, A. La Rosa, J. L. La Rosa Navarro, L. La Rotonda, F. La Ruffa, C. Lacasta, F. Lacava, J. Lacey, H. Lacker, D. Lacour, E. Ladygin, R. Lafaye, B. Laforge, T. Lagouri, S. Lai, S. Lammers, W. Lampl, E. Lançon, U. Landgraf, M. P. J. Landon, M. C. Lanfermann, V. S. Lang, J. C. Lange, R. J. Langenberg, A. J. Lankford, F. Lanni, K. Lantzsch, A. Lanza, A. Lapertosa, S. Laplace, J. F. Laporte, T. Lari, F. Lasagni Manghi, M. Lassnig, P. Laurelli, W. Lavrijsen, A. T. Law, P. Laycock, T. Lazovich, M. Lazzaroni, B. Le, O. Le Dortz, E. Le Guirriec, E. P. Le Quilleuc, M. LeBlanc, T. LeCompte, F. Ledroit-Guillon, C. A. Lee, G. R. Lee, S. C. Lee, L. Lee, B. Lefebvre, G. Lefebvre, M. Lefebvre, F. Legger, C. Leggett, G. Lehmann Miotto, X. Lei, W. A. Leight, M. A. L. Leite, R. Leitner, D. Lellouch, B. Lemmer, K. J. C. Leney, T. Lenz, B. Lenzi, R. Leone, S. Leone, C. Leonidopoulos, G. Lerner, C. Leroy, A. A. J. Lesage, C. G. Lester, M. Levchenko, J. Levêque, D. Levin, L. J. Levinson, M. Levy, D. Lewis, B. Li, C.-Q. Li, H. Li, L. Li, Q. Li, Q. Li, S. Li, X. Li, Y. Li, Z. Liang, B. Liberti, A. Liblong, K. Lie, J. Liebal, W. Liebig, A. Limosani, S. C. Lin, T. H. Lin, B. E. Lindquist, A. E. Lionti, E. Lipeles, A. Lipniacka, M. Lisovyi, T. M. Liss, A. Lister, A. M. Litke, B. Liu, H. Liu, H. Liu, J. K. K. Liu, J. Liu, J. B. Liu, K. Liu, L. Liu, M. Liu, Y. L. Liu, Y. Liu, M. Livan, A. Lleres, J. Llorente Merino, S. L. Lloyd, C. Y. Lo, F. Lo Sterzo, E. M. Lobodzinska, P. Loch, F. K. Loebinger, A. Loesle, K. M. Loew, A. Loginov, T. Lohse, K. Lohwasser, M. Lokajicek, B. A. Long, J. D. Long, R. E. Long, L. Longo, K. A. Looper, J. A. Lopez, D. Lopez Mateos, I. Lopez Paz, A. Lopez Solis, J. Lorenz, N. Lorenzo Martinez, M. Losada, P. J. Lösel, X. Lou, A. Lounis, J. Love, P. A. Love, H. Lu, N. Lu, Y. J. Lu, H. J. Lubatti, C. Luci, A. Lucotte, C. Luedtke, F. Luehring, W. Lukas, L. Luminari, O. Lundberg, B. Lund-Jensen, M. S. Lutz, P. M. Luzi, D. Lynn, R. Lysak, E. Lytken, F. Lyu, V. Lyubushkin, H. Ma, L. L. Ma, Y. Ma, G. Maccarrone, A. Macchiolo, C. M. Macdonald, B. Maček, J. Machado Miguens, D. Madaffari, R. Madar, W. F. Mader, A. Madsen, J. Maeda, S. Maeland, T. Maeno, A. S. Maevskiy, V. Magerl, J. Mahlstedt, C. Maiani, C. Maidantchik, A. A. Maier, T. Maier, A. Maio, O. Majersky, S. Majewski, Y. Makida, N. Makovec, B. Malaescu, Pa. Malecki, V. P. Maleev, F. Malek, U. Mallik, D. Malon, C. Malone, S. Maltezos, S. Malyukov, J. Mamuzic, G. Mancini, I. Mandić, J. Maneira, L. Manhaes de Andrade Filho, J. Manjarres Ramos, K. H. Mankinen, A. Mann, A. Manousos, B. Mansoulie, J. D. Mansour, R. Mantifel, M. Mantoani, S. Manzoni, L. Mapelli, G. Marceca, L. March, L. Marchese, G. Marchiori, M. Marcisovsky, M. Marjanovic, D. E. Marley, F. Marroquim, S. P. Marsden, Z. Marshall, M. U. F Martensson, S. Marti-Garcia, C. B. Martin, T. A. Martin, V. J. Martin, B. Martin dit Latour, M. Martinez, V. I. Martinez Outschoorn, S. Martin-Haugh, V. S. Martoiu, A. C. Martyniuk, A. Marzin, L. Masetti, T. Mashimo, R. Mashinistov, J. Masik, A. L. Maslennikov, L. Massa, P. Mastrandrea, A. Mastroberardino, T. Masubuchi, P. Mättig, J. Maurer, S. J. Maxfield, D. A. Maximov, R. Mazini, I. Maznas, S. M. Mazza, N. C. Mc Fadden, G. Mc Goldrick, S. P. Mc Kee, A. McCarn, R. L. McCarthy, T. G. McCarthy, L. I. McClymont, E. F. McDonald, J. A. Mcfayden, G. Mchedlidze, S. J. McMahon, P. C. McNamara, R. A. McPherson, S. Meehan, T. J. Megy, S. Mehlhase, A. Mehta, T. Meideck, K. Meier, B. Meirose, D. Melini, B. R. Mellado Garcia, J. D. Mellenthin, M. Melo, F. Meloni, A. Melzer, S. B. Menary, L. Meng, X. T. Meng, A. Mengarelli, S. Menke, E. Meoni, S. Mergelmeyer, P. Mermod, L. Merola, C. Meroni, F. S. Merritt, A. Messina, J. Metcalfe, A. S. Mete, C. Meyer, J-P. Meyer, J. Meyer, H. Meyer Zu Theenhausen, F. Miano, R. P. Middleton, S. Miglioranzi, L. Mijović, G. Mikenberg, M. Mikestikova, M. Mikuž, M. Milesi, A. Milic, D. W. Miller, C. Mills, A. Milov, D. A. Milstead, A. A. Minaenko, Y. Minami, I. A. Minashvili, A. I. Mincer, B. Mindur, M. Mineev, Y. Minegishi, Y. Ming, L. M. Mir, K. P. Mistry, T. Mitani, J. Mitrevski, V. A. Mitsou, A. Miucci, P. S. Miyagawa, A. Mizukami, J. U. Mjörnmark, T. Mkrtchyan, M. Mlynarikova, T. Moa, K. Mochizuki, P. Mogg, S. Mohapatra, S. Molander, R. Moles-Valls, R. Monden, M. C. Mondragon, K. Mönig, J. Monk, E. Monnier, A. Montalbano, J. Montejo Berlingen, F. Monticelli, S. Monzani, R. W. Moore, N. Morange, D. Moreno, M. Moreno Llácer, P. Morettini, S. Morgenstern, D. Mori, T. Mori, M. Morii, M. Morinaga, V. Morisbak, A. K. Morley, G. Mornacchi, J. D. Morris, L. Morvaj, P. Moschovakos, M. Mosidze, H. J. Moss, J. Moss, K. Motohashi, R. Mount, E. Mountricha, E. J. W. Moyse, S. Muanza, F. Mueller, J. Mueller, R. S. P. Mueller, D. Muenstermann, P. Mullen, G. A. Mullier, F. J. Munoz Sanchez, W. J. Murray, H. Musheghyan, M. Muškinja, A. G. Myagkov, M. Myska, B. P. Nachman, O. Nackenhorst, K. Nagai, R. Nagai, K. Nagano, Y. Nagasaka, K. Nagata, M. Nagel, E. Nagy, A. M. Nairz, Y. Nakahama, K. Nakamura, T. Nakamura, I. Nakano, R. F. Naranjo Garcia, R. Narayan, D. I. Narrias Villar, I. Naryshkin, T. Naumann, G. Navarro, R. Nayyar, H. A. Neal, P. Yu. Nechaeva, T. J. Neep, A. Negri, M. Negrini, S. Nektarijevic, C. Nellist, A. Nelson, M. E. Nelson, S. Nemecek, P. Nemethy, M. Nessi, M. S. Neubauer, M. Neumann, P. R. Newman, T. Y. Ng, T. Nguyen Manh, R. B. Nickerson, R. Nicolaidou, J. Nielsen, V. Nikolaenko, I. Nikolic-Audit, K. Nikolopoulos, J. K. Nilsen, P. Nilsson, Y. Ninomiya, A. Nisati, N. Nishu, R. Nisius, I. Nitsche, T. Nitta, T. Nobe, Y. Noguchi, M. Nomachi, I. Nomidis, M. A. Nomura, T. Nooney, M. Nordberg, N. Norjoharuddeen, O. Novgorodova, S. Nowak, M. Nozaki, L. Nozka, K. Ntekas, E. Nurse, F. Nuti, K. O’connor, D. C. O’Neil, A. A. O’Rourke, V. O’Shea, F. G. Oakham, H. Oberlack, T. Obermann, J. Ocariz, A. Ochi, I. Ochoa, J. P. Ochoa-Ricoux, S. Oda, S. Odaka, A. Oh, S. H. Oh, C. C. Ohm, H. Ohman, H. Oide, H. Okawa, Y. Okumura, T. Okuyama, A. Olariu, L. F. Oleiro Seabra, S. A. Olivares Pino, D. Oliveira Damazio, A. Olszewski, J. Olszowska, A. Onofre, K. Onogi, P. U. E. Onyisi, H. Oppen, M. J. Oreglia, Y. Oren, D. Orestano, N. Orlando, R. S. Orr, B. Osculati, R. Ospanov, G. Otero y Garzon, H. Otono, M. Ouchrif, F. Ould-Saada, A. Ouraou, K. P. Oussoren, Q. Ouyang, M. Owen, R. E. Owen, V. E. Ozcan, N. Ozturk, K. Pachal, A. Pacheco Pages, L. Pacheco Rodriguez, C. Padilla Aranda, S. Pagan Griso, M. Paganini, F. Paige, G. Palacino, S. Palazzo, S. Palestini, M. Palka, D. Pallin, E. St. Panagiotopoulou, I. Panagoulias, C. E. Pandini, J. G. Panduro Vazquez, P. Pani, S. Panitkin, D. Pantea, L. Paolozzi, Th. D. Papadopoulou, K. Papageorgiou, A. Paramonov, D. Paredes Hernandez, A. J. Parker, M. A. Parker, K. A. Parker, F. Parodi, J. A. Parsons, U. Parzefall, V. R. Pascuzzi, J. M. Pasner, E. Pasqualucci, S. Passaggio, Fr. Pastore, S. Pataraia, J. R. Pater, T. Pauly, B. Pearson, S. Pedraza Lopez, R. Pedro, S. V. Peleganchuk, O. Penc, C. Peng, H. Peng, J. Penwell, B. S. Peralva, M. M. Perego, D. V. Perepelitsa, F. Peri, L. Perini, H. Pernegger, S. Perrella, R. Peschke, V. D. Peshekhonov, K. Peters, R. F. Y. Peters, B. A. Petersen, T. C. Petersen, E. Petit, A. Petridis, C. Petridou, P. Petroff, E. Petrolo, M. Petrov, F. Petrucci, N. E. Pettersson, A. Peyaud, R. Pezoa, F. H. Phillips, P. W. Phillips, G. Piacquadio, E. Pianori, A. Picazio, E. Piccaro, M. A. Pickering, R. Piegaia, J. E. Pilcher, A. D. Pilkington, A. W. J. Pin, M. Pinamonti, J. L. Pinfold, H. Pirumov, M. Pitt, L. Plazak, M.-A. Pleier, V. Pleskot, E. Plotnikova, D. Pluth, P. Podberezko, R. Poettgen, R. Poggi, L. Poggioli, I. Pogrebnyak, D. Pohl, G. Polesello, A. Poley, A. Policicchio, R. Polifka, A. Polini, C. S. Pollard, V. Polychronakos, K. Pommès, D. Ponomarenko, L. Pontecorvo, G. A. Popeneciu, S. Pospisil, K. Potamianos, I. N. Potrap, C. J. Potter, T. Poulsen, J. Poveda, M. E. Pozo Astigarraga, P. Pralavorio, A. Pranko, S. Prell, D. Price, M. Primavera, S. Prince, N. Proklova, K. Prokofiev, F. Prokoshin, S. Protopopescu, J. Proudfoot, M. Przybycien, A. Puri, P. Puzo, J. Qian, G. Qin, Y. Qin, A. Quadt, M. Queitsch-Maitland, D. Quilty, S. Raddum, V. Radeka, V. Radescu, S. K. Radhakrishnan, P. Radloff, P. Rados, F. Ragusa, G. Rahal, J. A. Raine, S. Rajagopalan, C. Rangel-Smith, T. Rashid, S. Raspopov, M. G. Ratti, D. M. Rauch, F. Rauscher, S. Rave, I. Ravinovich, J. H. Rawling, M. Raymond, A. L. Read, N. P. Readioff, M. Reale, D. M. Rebuzzi, A. Redelbach, G. Redlinger, R. Reece, R. G. Reed, K. Reeves, L. Rehnisch, J. Reichert, A. Reiss, C. Rembser, H. Ren, M. Rescigno, S. Resconi, E. D. Resseguie, S. Rettie, E. Reynolds, O. L. Rezanova, P. Reznicek, R. Rezvani, R. Richter, S. Richter, E. Richter-Was, O. Ricken, M. Ridel, P. Rieck, C. J. Riegel, J. Rieger, O. Rifki, M. Rijssenbeek, A. Rimoldi, M. Rimoldi, L. Rinaldi, G. Ripellino, B. Ristić, E. Ritsch, I. Riu, F. Rizatdinova, E. Rizvi, C. Rizzi, R. T. Roberts, S. H. Robertson, A. Robichaud-Veronneau, D. Robinson, J. E. M. Robinson, A. Robson, E. Rocco, C. Roda, Y. Rodina, S. Rodriguez Bosca, A. Rodriguez Perez, D. Rodriguez Rodriguez, S. Roe, C. S. Rogan, O. Røhne, J. Roloff, A. Romaniouk, M. Romano, S. M. Romano Saez, E. Romero Adam, N. Rompotis, M. Ronzani, L. Roos, S. Rosati, K. Rosbach, P. Rose, N.-A. Rosien, E. Rossi, L. P. Rossi, J. H. N. Rosten, R. Rosten, M. Rotaru, J. Rothberg, D. Rousseau, A. Rozanov, Y. Rozen, X. Ruan, F. Rubbo, F. Rühr, A. Ruiz-Martinez, Z. Rurikova, N. A. Rusakovich, H. L. Russell, J. P. Rutherfoord, N. Ruthmann, Y. F. Ryabov, M. Rybar, G. Rybkin, S. Ryu, A. Ryzhov, G. F. Rzehorz, A. F. Saavedra, G. Sabato, S. Sacerdoti, H.F-W. Sadrozinski, R. Sadykov, F. Safai Tehrani, P. Saha, M. Sahinsoy, M. Saimpert, M. Saito, T. Saito, H. Sakamoto, Y. Sakurai, G. Salamanna, J. E. Salazar Loyola, D. Salek, P. H. Sales De Bruin, D. Salihagic, A. Salnikov, J. Salt, D. Salvatore, F. Salvatore, A. Salvucci, A. Salzburger, D. Sammel, D. Sampsonidis, D. Sampsonidou, J. Sánchez, V. Sanchez Martinez, A. Sanchez Pineda, H. Sandaker, R. L. Sandbach, C. O. Sander, M. Sandhoff, C. Sandoval, D. P. C. Sankey, M. Sannino, Y. Sano, A. Sansoni, C. Santoni, H. Santos, I. Santoyo Castillo, A. Sapronov, J. G. Saraiva, B. Sarrazin, O. Sasaki, K. Sato, E. Sauvan, G. Savage, P. Savard, N. Savic, C. Sawyer, L. Sawyer, J. Saxon, C. Sbarra, A. Sbrizzi, T. Scanlon, D. A. Scannicchio, M. Scarcella, J. Schaarschmidt, P. Schacht, B. M. Schachtner, D. Schaefer, L. Schaefer, R. Schaefer, J. Schaeffer, S. Schaepe, S. Schaetzel, U. Schäfer, A. C. Schaffer, D. Schaile, R. D. Schamberger, V. A. Schegelsky, D. Scheirich, M. Schernau, C. Schiavi, S. Schier, L. K. Schildgen, C. Schillo, M. Schioppa, S. Schlenker, K. R. Schmidt-Sommerfeld, K. Schmieden, C. Schmitt, S. Schmitt, S. Schmitz, U. Schnoor, L. Schoeffel, A. Schoening, B. D. Schoenrock, E. Schopf, M. Schott, J. F. P. Schouwenberg, J. Schovancova, S. Schramm, N. Schuh, A. Schulte, M. J. Schultens, H.-C. Schultz-Coulon, H. Schulz, M. Schumacher, B. A. Schumm, Ph. Schune, A. Schwartzman, T. A. Schwarz, H. Schweiger, Ph. Schwemling, R. Schwienhorst, J. Schwindling, A. Sciandra, G. Sciolla, M. Scornajenghi, F. Scuri, F. Scutti, J. Searcy, P. Seema, S. C. Seidel, A. Seiden, J. M. Seixas, G. Sekhniaidze, K. Sekhon, S. J. Sekula, N. Semprini-Cesari, S. Senkin, C. Serfon, L. Serin, L. Serkin, M. Sessa, R. Seuster, H. Severini, T. Sfiligoj, F. Sforza, A. Sfyrla, E. Shabalina, N. W. Shaikh, L. Y. Shan, R. Shang, J. T. Shank, M. Shapiro, P. B. Shatalov, K. Shaw, S. M. Shaw, A. Shcherbakova, C. Y. Shehu, Y. Shen, N. Sherafati, P. Sherwood, L. Shi, S. Shimizu, C. O. Shimmin, M. Shimojima, I. P. J. Shipsey, S. Shirabe, M. Shiyakova, J. Shlomi, A. Shmeleva, D. Shoaleh Saadi, M. J. Shochet, S. Shojaii, D. R. Shope, S. Shrestha, E. Shulga, M. A. Shupe, P. Sicho, A. M. Sickles, P. E. Sidebo, E. Sideras Haddad, O. Sidiropoulou, A. Sidoti, F. Siegert, Dj. Sijacki, J. Silva, S. B. Silverstein, V. Simak, Lj. Simic, S. Simion, E. Simioni, B. Simmons, M. Simon, P. Sinervo, N. B. Sinev, M. Sioli, G. Siragusa, I. Siral, S. Yu. Sivoklokov, J. Sjölin, M. B. Skinner, P. Skubic, M. Slater, T. Slavicek, M. Slawinska, K. Sliwa, R. Slovak, V. Smakhtin, B. H. Smart, J. Smiesko, N. Smirnov, S. Yu. Smirnov, Y. Smirnov, L. N. Smirnova, O. Smirnova, J. W. Smith, M. N. K. Smith, R. W. Smith, M. Smizanska, K. Smolek, A. A. Snesarev, I. M. Snyder, S. Snyder, R. Sobie, F. Socher, A. Soffer, A. Søgaard, D. A. Soh, G. Sokhrannyi, C. A. Solans Sanchez, M. Solar, E. Yu. Soldatov, U. Soldevila, A. A. Solodkov, A. Soloshenko, O. V. Solovyanov, V. Solovyev, P. Sommer, H. Son, A. Sopczak, D. Sosa, C. L. Sotiropoulou, R. Soualah, A. M. Soukharev, D. South, B. C. Sowden, S. Spagnolo, M. Spalla, M. Spangenberg, F. Spanò, D. Sperlich, F. Spettel, T. M. Spieker, R. Spighi, G. Spigo, L. A. Spiller, M. Spousta, R. D. St. Denis, A. Stabile, R. Stamen, S. Stamm, E. Stanecka, R. W. Stanek, C. Stanescu, M. M. Stanitzki, B. S. Stapf, S. Stapnes, E. A. Starchenko, G. H. Stark, J. Stark, S. H Stark, P. Staroba, P. Starovoitov, S. Stärz, R. Staszewski, P. Steinberg, B. Stelzer, H. J. Stelzer, O. Stelzer-Chilton, H. Stenzel, G. A. Stewart, M. C. Stockton, M. Stoebe, G. Stoicea, P. Stolte, S. Stonjek, A. R. Stradling, A. Straessner, M. E. Stramaglia, J. Strandberg, S. Strandberg, M. Strauss, P. Strizenec, R. Ströhmer, D. M. Strom, R. Stroynowski, A. Strubig, S. A. Stucci, B. Stugu, N. A. Styles, D. Su, J. Su, S. Suchek, Y. Sugaya, M. Suk, V. V. Sulin, DMS Sultan, S. Sultansoy, T. Sumida, S. Sun, X. Sun, K. Suruliz, C. J. E. Suster, M. R. Sutton, S. Suzuki, M. Svatos, M. Swiatlowski, S. P. Swift, I. Sykora, T. Sykora, D. Ta, K. Tackmann, J. Taenzer, A. Taffard, R. Tafirout, E. Tahirovic, N. Taiblum, H. Takai, R. Takashima, E. H. Takasugi, T. Takeshita, Y. Takubo, M. Talby, A. A. Talyshev, J. Tanaka, M. Tanaka, R. Tanaka, S. Tanaka, R. Tanioka, B. B. Tannenwald, S. Tapia Araya, S. Tapprogge, S. Tarem, G. F. Tartarelli, P. Tas, M. Tasevsky, T. Tashiro, E. Tassi, A. Tavares Delgado, Y. Tayalati, A. C. Taylor, G. N. Taylor, P. T. E. Taylor, W. Taylor, P. Teixeira-Dias, D. Temple, H. Ten Kate, P. K. Teng, J. J. Teoh, F. Tepel, S. Terada, K. Terashi, J. Terron, S. Terzo, M. Testa, R. J. Teuscher, T. Theveneaux-Pelzer, F. Thiele, J. P. Thomas, J. Thomas-Wilsker, P. D. Thompson, A. S. Thompson, L. A. Thomsen, E. Thomson, M. J. Tibbetts, R. E. Ticse Torres, V. O. Tikhomirov, Yu. A. Tikhonov, S. Timoshenko, P. Tipton, S. Tisserant, K. Todome, S. Todorova-Nova, S. Todt, J. Tojo, S. Tokár, K. Tokushuku, E. Tolley, L. Tomlinson, M. Tomoto, L. Tompkins, K. Toms, B. Tong, P. Tornambe, E. Torrence, H. Torres, E. Torró Pastor, J. Toth, F. Touchard, D. R. Tovey, C. J. Treado, T. Trefzger, F. Tresoldi, A. Tricoli, I. M. Trigger, S. Trincaz-Duvoid, M. F. Tripiana, W. Trischuk, B. Trocmé, A. Trofymov, C. Troncon, M. Trottier-McDonald, M. Trovatelli, L. Truong, M. Trzebinski, A. Trzupek, K. W. Tsang, J.C-L. Tseng, P. V. Tsiareshka, G. Tsipolitis, N. Tsirintanis, S. Tsiskaridze, V. Tsiskaridze, E. G. Tskhadadze, K. M. Tsui, I. I. Tsukerman, V. Tsulaia, S. Tsuno, D. Tsybychev, Y. Tu, A. Tudorache, V. Tudorache, T. T. Tulbure, A. N. Tuna, S. A. Tupputi, S. Turchikhin, D. Turgeman, I. Turk Cakir, R. Turra, P. M. Tuts, G. Ucchielli, I. Ueda, M. Ughetto, F. Ukegawa, G. Unal, A. Undrus, G. Unel, F. C. Ungaro, Y. Unno, C. Unverdorben, J. Urban, P. Urquijo, P. Urrejola, G. Usai, J. Usui, L. Vacavant, V. Vacek, B. Vachon, K. O. H. Vadla, A. Vaidya, C. Valderanis, E. Valdes Santurio, M. Valente, S. Valentinetti, A. Valero, L. Valéry, S. Valkar, A. Vallier, J. A. Valls Ferrer, W. Van Den Wollenberg, H. van der Graaf, P. van Gemmeren, J. Van Nieuwkoop, I. van Vulpen, M. C. van Woerden, M. Vanadia, W. Vandelli, A. Vaniachine, P. Vankov, G. Vardanyan, R. Vari, E. W. Varnes, C. Varni, T. Varol, D. Varouchas, A. Vartapetian, K. E. Varvell, J. G. Vasquez, G. A. Vasquez, F. Vazeille, T. Vazquez Schroeder, J. Veatch, V. Veeraraghavan, L. M. Veloce, F. Veloso, S. Veneziano, A. Ventura, M. Venturi, N. Venturi, A. Venturini, V. Vercesi, M. Verducci, W. Verkerke, A. T. Vermeulen, J. C. Vermeulen, M. C. Vetterli, N. Viaux Maira, O. Viazlo, I. Vichou, T. Vickey, O. E. Vickey Boeriu, G. H. A. Viehhauser, S. Viel, L. Vigani, M. Villa, M. Villaplana Perez, E. Vilucchi, M. G. Vincter, V. B. Vinogradov, A. Vishwakarma, C. Vittori, I. Vivarelli, S. Vlachos, M. Vogel, P. Vokac, G. Volpi, H. von der Schmitt, E. von Toerne, V. Vorobel, K. Vorobev, M. Vos, R. Voss, J. H. Vossebeld, N. Vranjes, M. Vranjes Milosavljevic, V. Vrba, M. Vreeswijk, R. Vuillermet, I. Vukotic, P. Wagner, W. Wagner, J. Wagner-Kuhr, H. Wahlberg, S. Wahrmund, J. Wakabayashi, J. Walder, R. Walker, W. Walkowiak, V. Wallangen, C. Wang, C. Wang, F. Wang, H. Wang, H. Wang, J. Wang, J. Wang, Q. Wang, R. Wang, S. M. Wang, T. Wang, W. Wang, W. Wang, Z. Wang, C. Wanotayaroj, A. Warburton, C. P. Ward, D. R. Wardrope, A. Washbrook, P. M. Watkins, A. T. Watson, M. F. Watson, G. Watts, S. Watts, B. M. Waugh, A. F. Webb, S. Webb, M. S. Weber, S. W. Weber, S. A. Weber, J. S. Webster, A. R. Weidberg, B. Weinert, J. Weingarten, M. Weirich, C. Weiser, H. Weits, P. S. Wells, T. Wenaus, T. Wengler, S. Wenig, N. Wermes, M. D. Werner, P. Werner, M. Wessels, T. D. Weston, K. Whalen, N. L. Whallon, A. M. Wharton, A. S. White, A. White, M. J. White, R. White, D. Whiteson, B. W. Whitmore, F. J. Wickens, W. Wiedenmann, M. Wielers, C. Wiglesworth, L. A. M. Wiik-Fuchs, A. Wildauer, F. Wilk, H. G. Wilkens, H. H. Williams, S. Williams, C. Willis, S. Willocq, J. A. Wilson, I. Wingerter-Seez, E. Winkels, F. Winklmeier, O. J. Winston, B. T. Winter, M. Wittgen, M. Wobisch, T. M. H. Wolf, R. Wolff, M. W. Wolter, H. Wolters, V. W. S. Wong, S. D. Worm, B. K. Wosiek, J. Wotschack, K. W. Wozniak, M. Wu, S. L. Wu, X. Wu, Y. Wu, T. R. Wyatt, B. M. Wynne, S. Xella, Z. Xi, L. Xia, D. Xu, L. Xu, T. Xu, B. Yabsley, S. Yacoob, D. Yamaguchi, Y. Yamaguchi, A. Yamamoto, S. Yamamoto, T. Yamanaka, M. Yamatani, K. Yamauchi, Y. Yamazaki, Z. Yan, H. Yang, H. Yang, Y. Yang, Z. Yang, W-M. Yao, Y. C. Yap, Y. Yasu, E. Yatsenko, K. H. Yau Wong, J. Ye, S. Ye, I. Yeletskikh, E. Yigitbasi, E. Yildirim, K. Yorita, K. Yoshihara, C. Young, C. J. S. Young, J. Yu, J. Yu, S. P. Y. Yuen, I. Yusuff, B. Zabinski, G. Zacharis, R. Zaidan, A. M. Zaitsev, N. Zakharchuk, J. Zalieckas, A. Zaman, S. Zambito, D. Zanzi, C. Zeitnitz, G. Zemaityte, A. Zemla, J. C. Zeng, Q. Zeng, O. Zenin, T. Ženiš, D. Zerwas, D. Zhang, F. Zhang, G. Zhang, H. Zhang, J. Zhang, L. Zhang, L. Zhang, M. Zhang, P. Zhang, R. Zhang, R. Zhang, X. Zhang, Y. Zhang, Z. Zhang, X. Zhao, Y. Zhao, Z. Zhao, A. Zhemchugov, B. Zhou, C. Zhou, L. Zhou, M. Zhou, M. Zhou, N. Zhou, C. G. Zhu, H. Zhu, J. Zhu, Y. Zhu, X. Zhuang, K. Zhukov, A. Zibell, D. Zieminska, N. I. Zimine, C. Zimmermann, S. Zimmermann, Z. Zinonos, M. Zinser, M. Ziolkowski, L. Živković, G. Zobernig, A. Zoccoli, R. Zou, M. zur Nedden, L. Zwalinski, ATLAS Collaboration

**Affiliations:** 1 Department of Physics, University of Adelaide, Adelaide, Australia; 2 Physics Department, SUNY Albany, Albany, NY USA; 3 Department of Physics, University of Alberta, Edmonton, AB Canada; 4 Department of Physics, Ankara University, Ankara, Turkey; 5 Istanbul Aydin University, Istanbul, Turkey; 6 Division of Physics, TOBB University of Economics and Technology, Ankara, Turkey; 7 LAPP, CNRS/IN2P3 and Université Savoie Mont Blanc, Annecy-le-Vieux, France; 8 High Energy Physics Division, Argonne National Laboratory, Argonne, IL USA; 9 Department of Physics, University of Arizona, Tucson, AZ USA; 10 Department of Physics, The University of Texas at Arlington, Arlington, TX USA; 11 Physics Department, National and Kapodistrian University of Athens, Athens, Greece; 12 Physics Department, National Technical University of Athens, Zografou, Greece; 13 Department of Physics, The University of Texas at Austin, Austin, TX USA; 14 Institute of Physics, Azerbaijan Academy of Sciences, Baku, Azerbaijan; 15 Institut de Física d’Altes Energies (IFAE), The Barcelona Institute of Science and Technology, Barcelona, Spain; 16 Institute of Physics, University of Belgrade, Belgrade, Serbia; 17 Department for Physics and Technology, University of Bergen, Bergen, Norway; 18 Physics Division, Lawrence Berkeley National Laboratory and University of California, Berkeley, CA USA; 19 Department of Physics, Humboldt University, Berlin, Germany; 20 Albert Einstein Center for Fundamental Physics and Laboratory for High Energy Physics, University of Bern, Bern, Switzerland; 21 School of Physics and Astronomy, University of Birmingham, Birmingham, UK; 22 Department of Physics, Bogazici University, Istanbul, Turkey; 23 Department of Physics Engineering, Gaziantep University, Gaziantep, Turkey; 24 Faculty of Engineering and Natural Sciences, Istanbul Bilgi University, Istanbul, Turkey; 25 Faculty of Engineering and Natural Sciences, Bahcesehir University, Istanbul, Turkey; 26 Centro de Investigaciones, Universidad Antonio Narino, Bogotá, Colombia; 27 INFN Sezione di Bologna, Bologna, Italy; 28 Dipartimento di Fisica e Astronomia, Università di Bologna, Bologna, Italy; 29 Physikalisches Institut, University of Bonn, Bonn, Germany; 30 Department of Physics, Boston University, Boston, MA USA; 31 Department of Physics, Brandeis University, Waltham, MA USA; 32 Universidade Federal do Rio De Janeiro COPPE/EE/IF, Rio de Janeiro, Brazil; 33 Electrical Circuits Department, Federal University of Juiz de Fora (UFJF), Juiz de Fora, Brazil; 34 Federal University of Sao Joao del Rei (UFSJ), Sao Joao del Rei, Brazil; 35 Instituto de Fisica, Universidade de Sao Paulo, São Paulo, Brazil; 36 Physics Department, Brookhaven National Laboratory, Upton, NY USA; 37 Transilvania University of Brasov, Brasov, Romania; 38 Horia Hulubei National Institute of Physics and Nuclear Engineering, Bucharest, Romania; 39 Department of Physics, Alexandru Ioan Cuza University of Iasi, Iasi, Romania; 40 National Institute for Research and Development of Isotopic and Molecular Technologies, Physics Department, Cluj Napoca, Romania; 41 University Politehnica Bucharest, Bucharest, Romania; 42 West University in Timisoara, Timisoara, Romania; 43 Departamento de Física, Universidad de Buenos Aires, Buenos Aires, Argentina; 44 Cavendish Laboratory, University of Cambridge, Cambridge, UK; 45 Department of Physics, Carleton University, Ottawa, ON Canada; 46 CERN, Geneva, Switzerland; 47 Enrico Fermi Institute, University of Chicago, Chicago, IL USA; 48 Departamento de Física, Pontificia Universidad Católica de Chile, Santiago, Chile; 49 Departamento de Física, Universidad Técnica Federico Santa María, Valparaiso, Chile; 50 Institute of High Energy Physics, Chinese Academy of Sciences, Beijing, China; 51 Department of Physics, Nanjing University, Nanjing, Jiangsu China; 52 Physics Department, Tsinghua University, Beijing, 100084 China; 53 Department of Modern Physics and State Key Laboratory of Particle Detection and Electronics, University of Science and Technology of China, Hefei, Anhui China; 54 School of Physics, Shandong University, Jinan, Shandong China; 55 Department of Physics and Astronomy, Key Laboratory for Particle Physics, Astrophysics and Cosmology, Ministry of Education; Shanghai Key Laboratory for Particle Physics and Cosmology, Shanghai Jiao Tong University, Shanghai (also at PKU-CHEP), Shanghai, China; 56 Université Clermont Auvergne, CNRS/IN2P3, LPC, Clermont-Ferrand, France; 57 Nevis Laboratory, Columbia University, Irvington, NY USA; 58 Niels Bohr Institute, University of Copenhagen, Copenhagen, Denmark; 59 INFN Gruppo Collegato di Cosenza, Laboratori Nazionali di Frascati, Frascati, Italy; 60 Dipartimento di Fisica, Università della Calabria, Rende, Italy; 61 Faculty of Physics and Applied Computer Science, AGH University of Science and Technology, Kraków, Poland; 62 Marian Smoluchowski Institute of Physics, Jagiellonian University, Kraków, Poland; 63 Institute of Nuclear Physics, Polish Academy of Sciences, Kraków, Poland; 64 Physics Department, Southern Methodist University, Dallas, TX USA; 65 Physics Department, University of Texas at Dallas, Richardson, TX USA; 66 DESY, Hamburg and Zeuthen, Germany; 67 Lehrstuhl für Experimentelle Physik IV, Technische Universität Dortmund, Dortmund, Germany; 68 Institut für Kern- und Teilchenphysik, Technische Universität Dresden, Dresden, Germany; 69 Department of Physics, Duke University, Durham, NC USA; 70 SUPA-School of Physics and Astronomy, University of Edinburgh, Edinburgh, UK; 71 INFN e Laboratori Nazionali di Frascati, Frascati, Italy; 72 Fakultät für Mathematik und Physik, Albert-Ludwigs-Universität, Freiburg, Germany; 73 Departement de Physique Nucleaire et Corpusculaire, Université de Genève, Geneva, Switzerland; 74 INFN Sezione di Genova, Genoa, Italy; 75 Dipartimento di Fisica, Università di Genova, Genoa, Italy; 76 E. Andronikashvili Institute of Physics, Iv. Javakhishvili Tbilisi State University, Tbilisi, Georgia; 77 High Energy Physics Institute, Tbilisi State University, Tbilisi, Georgia; 78 II Physikalisches Institut, Justus-Liebig-Universität Giessen, Giessen, Germany; 79 SUPA-School of Physics and Astronomy, University of Glasgow, Glasgow, UK; 80 II Physikalisches Institut, Georg-August-Universität, Göttingen, Germany; 81 Laboratoire de Physique Subatomique et de Cosmologie, Université Grenoble-Alpes, CNRS/IN2P3, Grenoble, France; 82 Laboratory for Particle Physics and Cosmology, Harvard University, Cambridge, MA USA; 83 Kirchhoff-Institut für Physik, Ruprecht-Karls-Universität Heidelberg, Heidelberg, Germany; 84 Physikalisches Institut, Ruprecht-Karls-Universität Heidelberg, Heidelberg, Germany; 85 Faculty of Applied Information Science, Hiroshima Institute of Technology, Hiroshima, Japan; 86 Department of Physics, The Chinese University of Hong Kong, Shatin, N.T. Hong Kong; 87 Department of Physics, The University of Hong Kong, Hong Kong, China; 88 Department of Physics and Institute for Advanced Study, The Hong Kong University of Science and Technology, Clear Water Bay, Kowloon, Hong Kong, China; 89 Department of Physics, National Tsing Hua University, Hsinchu, Taiwan; 90 Department of Physics, Indiana University, Bloomington, IN USA; 91 Institut für Astro- und Teilchenphysik, Leopold-Franzens-Universität, Innsbruck, Austria; 92 University of Iowa, Iowa City, IA USA; 93 Department of Physics and Astronomy, Iowa State University, Ames, IA USA; 94 Joint Institute for Nuclear Research, JINR Dubna, Dubna, Russia; 95 KEK, High Energy Accelerator Research Organization, Tsukuba, Japan; 96 Graduate School of Science, Kobe University, Kobe, Japan; 97 Faculty of Science, Kyoto University, Kyoto, Japan; 98 Kyoto University of Education, Kyoto, Japan; 99 Research Center for Advanced Particle Physics and Department of Physics, Kyushu University, Fukuoka, Japan; 100 Instituto de Física La Plata, Universidad Nacional de La Plata and CONICET, La Plata, Argentina; 101 Physics Department, Lancaster University, Lancaster, UK; 102 INFN Sezione di Lecce, Lecce, Italy; 103 Dipartimento di Matematica e Fisica, Università del Salento, Lecce, Italy; 104 Oliver Lodge Laboratory, University of Liverpool, Liverpool, UK; 105 Department of Experimental Particle Physics, Jožef Stefan Institute and Department of Physics, University of Ljubljana, Ljubljana, Slovenia; 106 School of Physics and Astronomy, Queen Mary University of London, London, UK; 107 Department of Physics, Royal Holloway University of London, Surrey, UK; 108 Department of Physics and Astronomy, University College London, London, UK; 109 Louisiana Tech University, Ruston, LA USA; 110 Laboratoire de Physique Nucléaire et de Hautes Energies, UPMC and Université Paris-Diderot and CNRS/IN2P3, Paris, France; 111 Fysiska institutionen, Lunds universitet, Lund, Sweden; 112 Departamento de Fisica Teorica C-15, Universidad Autonoma de Madrid, Madrid, Spain; 113 Institut für Physik, Universität Mainz, Mainz, Germany; 114 School of Physics and Astronomy, University of Manchester, Manchester, UK; 115 CPPM, Aix-Marseille Université and CNRS/IN2P3, Marseille, France; 116 Department of Physics, University of Massachusetts, Amherst, MA USA; 117 Department of Physics, McGill University, Montreal, QC Canada; 118 School of Physics, University of Melbourne, Victoria, Australia; 119 Department of Physics, The University of Michigan, Ann Arbor, MI USA; 120 Department of Physics and Astronomy, Michigan State University, East Lansing, MI USA; 121 INFN Sezione di Milano, Milan, Italy; 122 Dipartimento di Fisica, Università di Milano, Milan, Italy; 123 B.I. Stepanov Institute of Physics, National Academy of Sciences of Belarus, Minsk, Republic of Belarus; 124 Research Institute for Nuclear Problems of Byelorussian State University, Minsk, Republic of Belarus; 125 Group of Particle Physics, University of Montreal, Montreal, QC Canada; 126 P.N. Lebedev Physical Institute of the Russian Academy of Sciences, Moscow, Russia; 127 Institute for Theoretical and Experimental Physics (ITEP), Moscow, Russia; 128 National Research Nuclear University MEPhI, Moscow, Russia; 129 D.V. Skobeltsyn Institute of Nuclear Physics, M.V. Lomonosov Moscow State University, Moscow, Russia; 130 Fakultät für Physik, Ludwig-Maximilians-Universität München, Munich, Germany; 131 Max-Planck-Institut für Physik (Werner-Heisenberg-Institut), Munich, Germany; 132 Nagasaki Institute of Applied Science, Nagasaki, Japan; 133 Graduate School of Science and Kobayashi-Maskawa Institute, Nagoya University, Nagoya, Japan; 134 INFN Sezione di Napoli, Naples, Italy; 135 Dipartimento di Fisica, Università di Napoli, Naples, Italy; 136 Department of Physics and Astronomy, University of New Mexico, Albuquerque, NM USA; 137 Institute for Mathematics, Astrophysics and Particle Physics, Radboud University Nijmegen/Nikhef, Nijmegen, The Netherlands; 138 Nikhef National Institute for Subatomic Physics and University of Amsterdam, Amsterdam, The Netherlands; 139 Department of Physics, Northern Illinois University, DeKalb, IL USA; 140 Budker Institute of Nuclear Physics, SB RAS, Novosibirsk, Russia; 141 Department of Physics, New York University, New York, NY USA; 142 Ohio State University, Columbus, OH USA; 143 Faculty of Science, Okayama University, Okayama, Japan; 144 Homer L. Dodge Department of Physics and Astronomy, University of Oklahoma, Norman, OK USA; 145 Department of Physics, Oklahoma State University, Stillwater, OK USA; 146 Palacký University, RCPTM, Olomouc, Czech Republic; 147 Center for High Energy Physics, University of Oregon, Eugene, OR USA; 148 LAL, Univ. Paris-Sud, CNRS/IN2P3, Université Paris-Saclay, Orsay, France; 149 Graduate School of Science, Osaka University, Osaka, Japan; 150 Department of Physics, University of Oslo, Oslo, Norway; 151 Department of Physics, Oxford University, Oxford, UK; 152 INFN Sezione di Pavia, Pavia, Italy; 153 Dipartimento di Fisica, Università di Pavia, Pavia, Italy; 154 Department of Physics, University of Pennsylvania, Philadelphia, PA USA; 155 National Research Centre “Kurchatov Institute” B.P. Konstantinov Petersburg Nuclear Physics Institute, St. Petersburg, Russia; 156 INFN Sezione di Pisa, Pisa, Italy; 157 Dipartimento di Fisica E. Fermi, Università di Pisa, Pisa, Italy; 158 Department of Physics and Astronomy, University of Pittsburgh, Pittsburgh, PA USA; 159 Laboratório de Instrumentação e Física Experimental de Partículas-LIP, Lisbon, Portugal; 160 Faculdade de Ciências, Universidade de Lisboa, Lisbon, Portugal; 161 Department of Physics, University of Coimbra, Coimbra, Portugal; 162 Centro de Física Nuclear da Universidade de Lisboa, Lisbon, Portugal; 163 Departamento de Fisica, Universidade do Minho, Braga, Portugal; 164 Departamento de Fisica Teorica y del Cosmos and CAFPE, Universidad de Granada, Granada, Spain; 165 Dep Fisica and CEFITEC of Faculdade de Ciencias e Tecnologia, Universidade Nova de Lisboa, Caparica, Lisbon, Portugal; 166 Institute of Physics, Academy of Sciences of the Czech Republic, Prague, Czech Republic; 167 Czech Technical University in Prague, Prague, Czech Republic; 168 Charles University, Faculty of Mathematics and Physics, Prague, Czech Republic; 169 State Research Center Institute for High Energy Physics (Protvino), NRC KI, Protvino, Russia; 170 Particle Physics Department, Rutherford Appleton Laboratory, Didcot, UK; 171 INFN Sezione di Roma, Rome, Italy; 172 Dipartimento di Fisica, Sapienza Università di Roma, Rome, Italy; 173 INFN Sezione di Roma Tor Vergata, Rome, Italy; 174 Dipartimento di Fisica, Università di Roma Tor Vergata, Rome, Italy; 175 INFN Sezione di Roma Tre, Rome, Italy; 176 Dipartimento di Matematica e Fisica, Università Roma Tre, Rome, Italy; 177 Faculté des Sciences Ain Chock, Réseau Universitaire de Physique des Hautes Energies-Université Hassan II, Casablanca, Morocco; 178 Centre National de l’Energie des Sciences Techniques Nucleaires, Rabat, Morocco; 179 Faculté des Sciences Semlalia, Université Cadi Ayyad, LPHEA-Marrakech, Marrakech, Morocco; 180 Faculté des Sciences, Université Mohamed Premier and LPTPM, Oujda, Morocco; 181 Faculté des Sciences, Université Mohammed V, Rabat, Morocco; 182 DSM/IRFU (Institut de Recherches sur les Lois Fondamentales de l’Univers), CEA Saclay (Commissariat à l’Energie Atomique et aux Energies Alternatives), Gif-sur-Yvette, France; 183 Santa Cruz Institute for Particle Physics, University of California Santa Cruz, Santa Cruz, CA USA; 184 Department of Physics, University of Washington, Seattle, WA USA; 185 Department of Physics and Astronomy, University of Sheffield, Sheffield, UK; 186 Department of Physics, Shinshu University, Nagano, Japan; 187 Department Physik, Universität Siegen, Siegen, Germany; 188 Department of Physics, Simon Fraser University, Burnaby, BC Canada; 189 SLAC National Accelerator Laboratory, Stanford, CA USA; 190 Faculty of Mathematics, Physics and Informatics, Comenius University, Bratislava, Slovak Republic; 191 Department of Subnuclear Physics, Institute of Experimental Physics of the Slovak Academy of Sciences, Kosice, Slovak Republic; 192 Department of Physics, University of Cape Town, Cape Town, South Africa; 193 Department of Physics, University of Johannesburg, Johannesburg, South Africa; 194 School of Physics, University of the Witwatersrand, Johannesburg, South Africa; 195 Department of Physics, Stockholm University, Stockholm, Sweden; 196 The Oskar Klein Centre, Stockholm, Sweden; 197 Physics Department, Royal Institute of Technology, Stockholm, Sweden; 198 Departments of Physics and Astronomy and Chemistry, Stony Brook University, Stony Brook, NY USA; 199 Department of Physics and Astronomy, University of Sussex, Brighton, UK; 200 School of Physics, University of Sydney, Sydney, Australia; 201 Institute of Physics, Academia Sinica, Taipei, Taiwan; 202 Department of Physics, Technion: Israel Institute of Technology, Haifa, Israel; 203 Raymond and Beverly Sackler School of Physics and Astronomy, Tel Aviv University, Tel Aviv, Israel; 204 Department of Physics, Aristotle University of Thessaloniki, Thessaloníki, Greece; 205 International Center for Elementary Particle Physics and Department of Physics, The University of Tokyo, Tokyo, Japan; 206 Graduate School of Science and Technology, Tokyo Metropolitan University, Tokyo, Japan; 207 Department of Physics, Tokyo Institute of Technology, Tokyo, Japan; 208 Tomsk State University, Tomsk, Russia; 209 Department of Physics, University of Toronto, Toronto, ON Canada; 210 INFN-TIFPA, Trento, Italy; 211 University of Trento, Trento, Italy; 212 TRIUMF, Vancouver, BC Canada; 213 Department of Physics and Astronomy, York University, Toronto, ON Canada; 214 Faculty of Pure and Applied Sciences, and Center for Integrated Research in Fundamental Science and Engineering, University of Tsukuba, Tsukuba, Japan; 215 Department of Physics and Astronomy, Tufts University, Medford, MA USA; 216 Department of Physics and Astronomy, University of California Irvine, Irvine, CA USA; 217 INFN Gruppo Collegato di Udine, Sezione di Trieste, Udine, Italy; 218 ICTP, Trieste, Italy; 219 Dipartimento di Chimica, Fisica e Ambiente, Università di Udine, Udine, Italy; 220 Department of Physics and Astronomy, University of Uppsala, Uppsala, Sweden; 221 Department of Physics, University of Illinois, Urbana, IL USA; 222 Instituto de Fisica Corpuscular (IFIC), Centro Mixto Universidad de Valencia-CSIC, Valencia, Spain; 223 Department of Physics, University of British Columbia, Vancouver, BC Canada; 224 Department of Physics and Astronomy, University of Victoria, Victoria, BC Canada; 225 Department of Physics, University of Warwick, Coventry, UK; 226 Waseda University, Tokyo, Japan; 227 Department of Particle Physics, The Weizmann Institute of Science, Rehovot, Israel; 228 Department of Physics, University of Wisconsin, Madison, WI USA; 229 Fakultät für Physik und Astronomie, Julius-Maximilians-Universität, Würzburg, Germany; 230 Fakultät für Mathematik und Naturwissenschaften, Fachgruppe Physik, Bergische Universität Wuppertal, Wuppertal, Germany; 231 Department of Physics, Yale University, New Haven, CT USA; 232 Yerevan Physics Institute, Yerevan, Armenia; 233 Centre de Calcul de l’Institut National de Physique Nucléaire et de Physique des Particules (IN2P3), Villeurbanne, France; 234 Academia Sinica Grid Computing, Institute of Physics, Academia Sinica, Taipei, Taiwan; 235 CERN, 1211 Geneva 23, Switzerland

## Abstract

This paper presents a study of $WW\gamma$ and $WZ\gamma$ triboson production using events from proton–proton collisions at a centre-of-mass energy of $\sqrt{s} = \text{8}\,\text{TeV}$ recorded with the ATLAS detector at the LHC and corresponding to an integrated luminosity of 20.2 fb$^{-1}$. The $WW\gamma$ production cross-section is determined using a final state containing an electron, a muon, a photon, and neutrinos ($e\nu \mu \nu \gamma$). Upper limits on the production cross-section of the $e\nu \mu \nu \gamma$ final state and the $WW\gamma$ and $WZ\gamma$ final states containing an electron or a muon, two jets, a photon, and a neutrino ($e\nu jj\gamma$ or $\mu \nu jj\gamma$) are also derived. The results are compared to the cross-sections predicted by the Standard Model at next-to-leading order in the strong-coupling constant. In addition, upper limits on the production cross-sections are derived in a fiducial region optimised for a search for new physics beyond the Standard Model. The results are interpreted in the context of anomalous quartic gauge couplings using an effective field theory. Confidence intervals at 95% confidence level are derived for the 14 coupling coefficients to which $WW\gamma$ and $WZ\gamma$ production are sensitive.

## Introduction

Measuring triboson final states at the Large Hadron Collider (LHC) [1] provides a test of the non-Abelian structure of the electroweak sector of the Standard Model (SM) of particle physics that predicts quartic gauge couplings. Deviations from the SM can be parametrised in the framework of anomalous quartic gauge couplings (aQGCs). This paper describes a measurement of $WV \gamma$ production by analysing events containing a $W$ boson, a vector boson ($V$), being either another $W$  boson or a $Z$  boson, and a photon, using proton–proton collisions at a centre-of-mass energy of $\sqrt{s} = 8\,\text {TeV}$ corresponding to an integrated luminosity of 20.2 fb$^{-1}$ recorded by the ATLAS detector [2].

At LEP, $WW\gamma$ production was studied at centre-of-mass energies ranging from 183 to 207 $\text {GeV}$ in a variety of photon plus leptonic or hadronic final states [3]. The analysis presented here has a higher energy reach than the results obtained at LEP. The production of $WV \gamma$ events was studied by the CMS Collaboration in Ref. [4] in final states containing electrons or muons and jets, and using a data set with a similar luminosity and the same centre-of-mass energy as employed here. Other analyses with three bosons in the final state and also sensitive to quartic gauge couplings have been performed by the ATLAS and the CMS collaborations [5–8]. Furthermore, exclusion limits on new physics beyond the SM described by aQGCs have also been set at the LHC using diboson final states including photons [9–11] and in diboson final states including massive gauge bosons only [12–17].

**Fig. 1:**
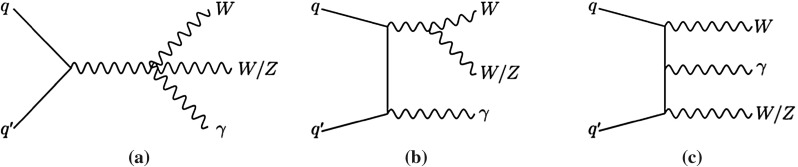
Examples of Feynman diagrams of $WV \gamma$ production at the LHC. In **a** the quartic vertex is shown, while **b**, **c** depict the production from radiative processes

In proton–proton collisions, $WV \gamma$ events are produced through the $WWZ\gamma$ and $WW\gamma \gamma$ quartic couplings as depicted in Fig. 1a or through radiation of one or more bosons as exemplified in Fig. 1b, c. The fully leptonic final state ($e\nu \mu \nu \gamma$) of $WW\gamma$ production containing an electron (*e*), a muon ($\mu$), their corresponding neutrinos ($\nu$), and a photon is studied as it has a clean experimental signature. The same-flavour final states, $e\nu e\nu \gamma$ and $\mu \nu \mu \nu \gamma$, are not studied as they have large backgrounds. Semileptonic final states ($\ell \nu jj\gamma$) containing one light lepton ($\ell = e \text { or } \mu$), a neutrino, two jets (*j*), and a photon are also studied. The analysis of the latter profits from the larger hadronic branching ratio of $W$- and $Z$-boson decays and is performed separately in the electron ($e\nu jj\gamma$) and the muon ($\mu \nu jj\gamma$) channels. The production of $WV \gamma$ events whose decays include $\tau$ leptons is not considered as signal.

Two fiducial regions are defined for all final states: one is optimised for the observation of the process while the other is optimised for a search for new physics beyond the SM. The results obtained in the latter region are interpreted in the context of aQGCs that describe modified triboson production using an effective field theory [18].

This paper is structured as follows. The ATLAS detector and the data employed in this analysis are described in Sect. 2. Section 3 details the Monte Carlo simulations used. The reconstruction of the detector information is outlined in Sect. 4. The analysis of the fully leptonic final state is described in Sect. 5 followed by the description of the semileptonic analysis in Sect. 6. In Sect. 7 the fiducial region of the cross-section measurement is defined and the determination of the production cross-section in the $e\nu \mu \nu \gamma$ final state is described. The derivation of upper limits on the $WV \gamma$ production cross-section is also presented. Section 8 discusses the cross-section exclusion limits in the fiducial region optimised for new physics beyond the SM and the interpretation of the results in the framework of aQGCs. A summary of the results is given in Sect. 9.

## ATLAS detector and data sample

The ATLAS experiment [2] at the LHC is a multipurpose particle detector with a forward-backward symmetric cylindrical geometry and a near $4\pi$ coverage in solid angle.^1^ It consists of an inner tracking detector surrounded by a thin superconducting solenoid providing a 2 T axial magnetic field, electromagnetic and hadronic calorimeters, and a muon spectrometer. The inner tracking detector covers the pseudorapidity range $|\eta | < 2.5$ and consists of silicon pixel, silicon microstrip, and transition radiation tracking detectors. Lead/liquid-argon (LAr) sampling calorimeters provide electromagnetic energy measurements with high granularity in the $\eta$–$\phi$ plane and a threefold segmentation in the radial direction. The first of the three layers of the LAr calorimeter has the smallest $\eta$-segmentation to discriminate between single photon showers and two overlapping showers coming from the decays of neutral hadrons. A hadronic (steel/scintillator-tile) calorimeter covers the central pseudorapidity range. The endcap and forward regions are instrumented with LAr calorimeters for the energy measurement of electromagnetic and hadronic showers up to $|\eta | = 4.9$. The muon spectrometer encompasses the calorimeters and includes a system of precision tracking chambers as well as fast detectors for triggering. It comprises three large air-core toroidal superconducting magnets with eight coils each. The field integral of the toroids ranges between 2.0 and 6.0 Tm across most of the detector. A three-level trigger system is used to select events for read-out and storage. The first-level trigger is implemented in hardware and uses a subset of the detector information to reduce the accepted rate to 75 KHz. This is followed by two software-based trigger levels that together reduce the accepted event rate to 400 Hz on average.

This analysis uses data recorded at a centre-of-mass energy of ${8\,\text {TeV}}$, corresponding to an integrated luminosity of $20.2\,\pm \,0.4\,\text {fb}^{-1}$ [19] after applying basic data quality criteria to ensure the full functionality of all detector subcomponents. Only events that have at least three reconstructed tracks [20] with $p_{\text {T}} > 500\,\text {MeV}$ associated with the primary vertex are considered for analysis. The primary vertex is defined as the vertex whose associated tracks have the largest sum of squared transverse momenta. Furthermore, events are discarded if they contain jets that are likely to be mismeasured.

Dedicated triggers are used for each final state. The events of the fully leptonic analysis are triggered by requiring three particles in the event: a muon with a transverse momentum ($p_{\text {T}}$) of at least 18 $\text {GeV}$ and two clusters of energy deposits in the electromagnetic calorimeter with a transverse energy ($E_{\text {T}}$) of at least 10 $\text {GeV}$. The efficiency of this trigger for the selection of the signal described in Sect. 5 corresponds to $0.82 \pm 0.01$(stat.). For the semileptonic final states, a combination of single-lepton triggers [21] is used to maintain a high efficiency over a wide range of lepton transverse momenta. The $e\nu jj\gamma$ final state is triggered by either requiring an isolated electron with $p_{\text {T}} > {24\,\text {GeV}}$ or an electron with $p_{\text {T}} > {60\,\text {GeV}}$ and no requirement on isolation. The lepton isolation is based on the sum of the transverse momenta of additional tracks in a cone of size $\Delta R = 0.2$ around the lepton’s track. This trigger combination provides an efficiency of $0.964 \pm 0.004$(stat.) for the signal selection described in Sect. 6. Similarly, the $\mu \nu jj\gamma$ final state is triggered by either requiring an isolated muon with $p_{\text {T}} > {24\,\text {GeV}}$ or a muon with $p_{\text {T}} > {36\,\text {GeV}}$ and no requirement on isolation. The efficiency of this trigger combination for the signal corresponds to $0.772 \pm 0.007$(stat.).

## Monte Carlo simulations

The expected signal and background events were simulated with Monte Carlo (MC) event generators. The simulations were used to optimise the selection criteria, to compute efficiencies, and to estimate the contributions of specific background processes. For the simulation of the MC samples, the ATLAS simulation infrastructure [22], which uses the GEANT4 toolkit [23] for the detector simulation, was employed. All simulations described in this section were computed at leading order (LO) in the perturbative expansion of the strong-coupling constant ($\alpha _{\text {S}}$) unless otherwise stated.

The $WV \gamma$ signal process was simulated with the MC event generator SHERPA 2.1.1 [24–27] with up to one additional parton in the matrix element, using the default tunes. The CT10NLO [28] set of parton distribution functions (PDF) was used. These signal predictions were normalised using the cross-sections of the fiducial regions introduced in Sect. 7, computed at next-to-leading order (NLO) in $\alpha _{\text {S}}$ using the VBFNLO 2.7.1 [29–32] program and the CT14NLO [33] PDF set. The renormalisation and factorisation scales were set to the invariant mass of the triboson system. The $WV \gamma$ processes that contain $\tau$ leptons in their decay are considered as background in this analysis and were simulated like the signal as just described. For cross-checks and for the estimation of systematic uncertainties associated with the event generation, the $WV \gamma$ signal process was also simulated using the MadGraph 5.2.2.2 [34] event generator with dynamical renormalisation and factorisation scales. It was interfaced to the PYTHIA 6.427 [35] program for the hadronisation and underlying event simulation with the Perugia 2012 [36] tune and used the CTEQ6L1 [37] PDF set. In addition, five reference samples modelling anomalous quartic gauge couplings were simulated for each studied final state, using the MadGraph event generator as described above and normalised using the corresponding cross-section predictions obtained at NLO with the VBFNLO program.

Backgrounds from *WZ*, *ZZ*, and $Z\gamma$ diboson production were simulated with up to three additional partons in the final state using the SHERPA event generator (versions 1.4.1, 1.4.5, and 1.4.1 with the default tunes respectively) with the CT10NLO PDF set. Top quark pair production in association with a photon ($t\bar{t}\gamma$) was generated with the MadGraph 5.2.1.0 event generator using the CTEQ6L1 PDF set and interfaced to PYTHIA 8.183 [38] for the simulation of the hadronisation and the underlying event using the AUET2B [39] tune. The cross-section was normalised using the computations of Ref. [40] which were performed at NLO in $\alpha _{\text {S}}$. The simultaneous production of top and antitop quarks ($t\bar{t}$) and the production of $W$ bosons in association with top quarks (*Wt*) were generated at NLO in $\alpha _{\text {S}}$ with the POWHEG-BOX [41–43] program using the CT10f4 PDF set and being interfaced to PYTHIA 6.426 with the Perugia 2011C [36] tune and using the CTEQ6L1 PDF set. The background from $Z$ bosons produced in association with jets ($Z$ + jets) and from $W$-boson production in association with a photon ($W\gamma$ + jets) were generated with the ALPGEN [44] program interfaced to the HERWIG 6.520.2 [45] event generator for parton showering and hadronisation and to the JIMMY [46] event generator to simulate the underlying event. The AUET2 [47] tune and the CTEQ6L1 PDF set were employed. All simulations that used the PYTHIA event generator employed the TAUOLA [48] program to compute the $\tau$ lepton decays. In samples that do not contain a prompt photon in the final state, the PHOTOS [49] program was employed to simulate photon radiation from final-state charged particles.

Contributions from additional proton–proton collisions accompanying the hard-scatter interaction, termed pile-up, were simulated using the PYTHIA 8.160 event generator. The resulting distribution of the mean number of interactions per bunch crossing was corrected to reproduce the distribution measured in data. The level of agreement between simulated and recorded data was further improved by correcting the simulated vertex distribution, object trigger and identification efficiencies, resolution and calibration to agree with the measured values [50–52].

## Event reconstruction

The selection of the $WV \gamma$ signal events is based on objects that are reconstructed using the same algorithms for simulated and recorded events. The reconstruction of electron and photon candidates employs energy clusters [53] of the calorimeters and their matching to tracks from the inner detector [50, 54]. The measured energies of the electrons and photons are corrected as described in Ref. [55]. Electron or photon candidates reconstructed within $1.37< |\eta | < 1.52$ are discarded as this corresponds to a transition region between different calorimeter components which has poor energy resolution and identification efficiencies for these objects.

Photon candidates are reconstructed within $|\eta | < 2.37$ and their transverse energy has to exceed 15 $\text {GeV}$. They are required to fulfil the *tight* identification criteria described in Ref. [51]. An isolation requirement is applied to reject hadronic backgrounds: the additional transverse energy deposited in the calorimeter in a cone of size $\Delta R = 0.4$ around the photon candidate, called $E_{\text {T}}^{\text {iso}}$, must be less than 4 $\text {GeV}$ after the median energy density of the event scaled to the cone size is subtracted in order to reduce the effect from pile-up [56].

Electron candidates are reconstructed within $|\eta | < 2.47$ and their transverse momentum has to exceed 7 $\text {GeV}$. They are required to fulfil the *tight* identification criteria described in Ref. [50]. In the fully leptonic analysis the same isolation requirement used for photons is applied to electrons as this facilitates the background estimation with the two-dimensional sideband method (see Sect. 5). The semileptonic analysis imposes a different isolation requirement, as it relies on other background estimation methods (see Sect. 6). For this analysis, the additional transverse energy deposited in the calorimeter in a cone of size $\Delta R = 0.3$ around the electron is required to be less than 14% of the transverse energy of the electron after the pile-up energy is subtracted as for the photons. Furthermore, a track-based isolation requirement is imposed: the sum of the transverse momenta of the additional tracks in the aforementioned cone is required to be less than 7% of the transverse energy of the electron itself. In addition, the semileptonic analysis requires the electron track to be consistent with coming from the primary vertex.

Muon candidates are reconstructed within $|\eta | < 2.4$ by combining tracks in the inner detector with tracks in the muon spectrometer. A statistical combination of the track parameters or a global refit of the tracks, described as *Chain 3* in Ref. [52], is used. Muon candidates are required to have a transverse momentum larger than 7 $\text {GeV}$ and to originate from the primary vertex. A track-based isolation requirement is imposed: the sum of the transverse momenta of the additional tracks in a cone of size $\Delta R = 0.2$ around the muon candidate is required to be less than 10% of the transverse momentum of the muon candidate itself.

Jet candidates are reconstructed within $|y| < 4.4$ from topological energy clusters [57] using the anti-$k_{t}$ algorithm [58] with a radius parameter of $R = 0.4$ implemented in the FastJet software package [59]. The measured energies of the jet candidates are corrected to the hadronic scale using the local cell signal weighting scheme [60] and their transverse momentum has to exceed 25 $\text {GeV}$. For central jets ($|\eta | < 2.4$) with $p_{\text {T}} < {50\,\text {GeV}}$, the scalar sum of the transverse momenta of tracks associated with the jet and originating from the primary vertex of the interaction is required to be at least 50% of the jet $p_{\text {T}}$. This requirement suppresses jets originating from pile-up interactions [61].

The possible overlap between the object candidates is removed by applying the following requirements sequentially. Any electron that lies within a cone of size $\Delta R = 0.1$ around a more energetic electron candidate or a muon candidate is discarded. Photon candidates are rejected if their angular distance to any remaining electron or muon is smaller than $\Delta R = 0.5$. Apart from the removal of overlapping objects, this requirement also suppresses photons that are radiated from the lepton in the final state. Jets are discarded if they lie within a cone of size $\Delta R = 0.3$ around an electron or $\Delta R = 0.5$ around a photon candidate. Finally, muon candidates are rejected if their angular distance to a jet is smaller than $\Delta R = 0.3$ in order to remove muons originating from heavy-flavour quark decays within jets.

The missing transverse momentum vector ($\vec {p}_{\text {T}}^{\text {\,miss}}$) of an event is a measure of the momentum imbalance in the transverse plane. It is calculated as the negative vector sum of the transverse momenta of calibrated leptons, photons, and jets, and additional tracks from the primary vertex that are not associated with any of those objects [62]. The missing transverse momentum ($E_{\text {T}}^{\text {miss}}$) is defined as the magnitude of $\vec {p}_{\text {T}}^{\text {\,miss}}$.

The missing transverse momentum is employed for the definition of the selection criteria of the semileptonic analysis described in Sect. 6. In the fully leptonic analysis, described in Sect. 5, the relative missing transverse momentum ($E_{\text {T,\,rel}}^{\text {miss}}$) is used as this improves the signal significance. Its definition is based on the absolute azimuthal separation ($\Delta \phi$) of the object closest to $\vec {p}_{\text {T}}^{\text {\,miss}}$:

1 $$ E_{\text {T,\,rel}}^{\text {miss}} = {\left\{ \begin{array}{ll} E_{\text {T}}^{\text {miss}} \times \sin (\Delta \phi ),&\, \text {if } \Delta \phi (\vec {p}_{\text {T}}^{\text {\,miss}},\text {closest object}) < \frac{\pi }{2}, \\ E_{\text {T}}^{\text {miss}},& \,\text {otherwise}. \end{array}\right. } $$

The transverse mass ($m_{\text {T}}$) is defined using $E_{\text {T}}^{\text {miss}}$, the transverse momentum ($p_{\text {T}}^{\ell }$) of the most energetic lepton in the event and the absolute angular difference between $\vec {p}_{\text {T}}^{\text {\,miss}}$ and this lepton ($\Delta \phi (\vec {p}_{\text {T}}^{\text {\,miss}}, \ell )$):

2 $$\begin{aligned} m_{\text {T}} = \sqrt{2 p_{\text {T}}^{\ell } E_{\text {T}}^{\text {miss}} [1- \cos (\Delta \phi (\vec {p}_{\text {T}}^{\text {\,miss}}, \ell ))]}. \end{aligned}$$

## Analysis of fully leptonic final states

In the fully leptonic analysis, $WW\gamma$ events are studied solely in the $e\nu \mu \nu \gamma$ final state. Events where the two $W$ bosons decay to leptons of the same flavour, i.e. $e\nu e\nu \gamma$ or $\mu \nu \mu \nu \gamma$ final states, have large backgrounds from Drell–Yan processes with photon radiation ($Z\gamma$) and do not increase the sensitivity of this measurement.

The event selection for the fully leptonic analysis requires the presence of exactly one electron and one muon with opposite electric charge, each with a transverse momentum of at least 20 $\text {GeV}$, at least one reconstructed photon with $E_{\text {T}} > {15\,\text {GeV}}$, and relative missing transverse momentum larger than 15 $\text {GeV}$. Events containing a third reconstructed electron or muon with $p_{\text {T}} > {7\,\text {GeV}}$ are discarded to suppress backgrounds from *WW* and *WZ* diboson production. For the rejection of Drell–Yan background decaying to $\tau$ leptons, the invariant mass of the electron–muon pair is required to be larger than 50 $\text {GeV}$. Finally, events containing any reconstructed jet with $p_{\text {T}} > {25\,\text {GeV}}$ are discarded, thereby reducing background contributions from top-quark production. These selection requirements are optimised to yield the best sensitivity to the signal and define the signal region. The expected number of signal events is $12.2 \pm 1.1$, as computed with the VBFNLO program and corrected for acceptance and efficiency effects (described in Sect. 7 along with the corresponding uncertainties). A total of 26 events are observed.

**Table 1:** Expected and observed event yields for the fully leptonic final state in the $e\nu \mu \nu \gamma$ signal region. For each background process the corresponding estimation method is stated along with the resulting event yield. The quoted uncertainties include statistical and systematic uncertainties. The uncertainty in the total background expectation is symmetrised. The expected signal is computed with the VBFNLO program and corrected for acceptance and efficiency

Process	Events	Estimation method
$t\bar{t}\gamma$	4.1 ± 1.9	MC simulation
$Z\gamma$	2.7 ± 1.2	MC simulation
$WZ\gamma$	2.7 ± 0.6	MC simulation
Fake $\gamma$ from *e*	2.3 ± 0.6	Corrected simulation
Fake $\gamma$ from jets	$1.7\ ^{+\,3.3}_{-\,1.4}$	2D sideband method
$WW\gamma$ ($\tau$ contribution)	1.0 ± 0.1	MC simulation
*Wt*	0.3 ± 0.1	MC simulation
*ZZ*	0.2 ± 0.1	MC simulation
Fake $\mu$ from jets	0.1 ± 0.1	MC simulation
Fake *e* from jets	$0.0\ ^{+\,0.6}_{-\,0.0}$	2D sideband method
Total background	15.1 ± 4.1	Sum of components
Expected signal	12.2 ± 1.1	Corrected VBFNLO
Data	26	Measurement

Several processes are backgrounds to the fully leptonic $WW\gamma$ signal; their contributions in the signal region are summarised in Table 1. The dominant source of background is the production of $t\bar{t}\gamma$ events where the top quarks decay to $W$ bosons and *b*-quarks with a leptonic decay of the $W$ boson ($t \rightarrow Wb \rightarrow \ell \nu b$). This process mimics the signal when the jets have low energy or are produced in the forward direction ($|y| \ge 4.4$) and hence the jets are not reconstructed. Other subdominant backgrounds are $Z\gamma$ events, which contribute when the $Z$ boson decays to a pair of leptonically decaying $\tau$ leptons, and $WZ\gamma$ production, which can mimic the signal when one of the final state leptons does not fulfil the identification criteria or is not reconstructed due to the limited geometrical acceptance. Other backgrounds arise from $WW\gamma$ production including $\tau$ leptons and the production of *Wt* and *ZZ* events. The event yields of all these processes are estimated using MC simulation. The corresponding uncertainties include statistical and systematic uncertainties that are of similar size. The systematic uncertainties can be subdivided into experimental uncertainties and uncertainties from the theoretical calculation. The two components contribute equally to the uncertainty for most processes. The relative uncertainties from the theoretical calculation range from 5 to 22% [6, 40, 63–66]; the uncertainties associated with the computation of the $WV \gamma$ process are described in Sect. 7. The experimental uncertainties include the energy scale and energy resolution uncertainties of the reconstructed objects [52, 55, 60, 67, 68], the uncertainties associated with the efficiencies of their reconstruction and identification [50, 52, 54], as well as uncertainties attributed to the simulation of the event pile-up [61]. The relative experimental uncertainties range from 5 to 32% with the largest contribution arising from the jet energy scale uncertainty which mainly contributes due to the requirement that the signal events should not contain reconstructed jets.

Events containing misidentified objects also constitute an important source of background. The background from *WZ* production where an electron is reconstructed as a photon (fake $\gamma$ from *e*) is estimated by using MC simulation, where the rate of electrons being reconstructed as photons is corrected to better describe the data. This rate is determined by studying the decays of $Z$ bosons to two electrons where one of the electrons is reconstructed as a photon and is below 6% for most of the pseudorapidity region. The uncertainty of this correction is small compared to the total uncertainty, which also includes the statistical uncertainty, uncertainties from the theoretical calculation, and experimental uncertainties as discussed in the previous paragraph.

**Fig. 2:**
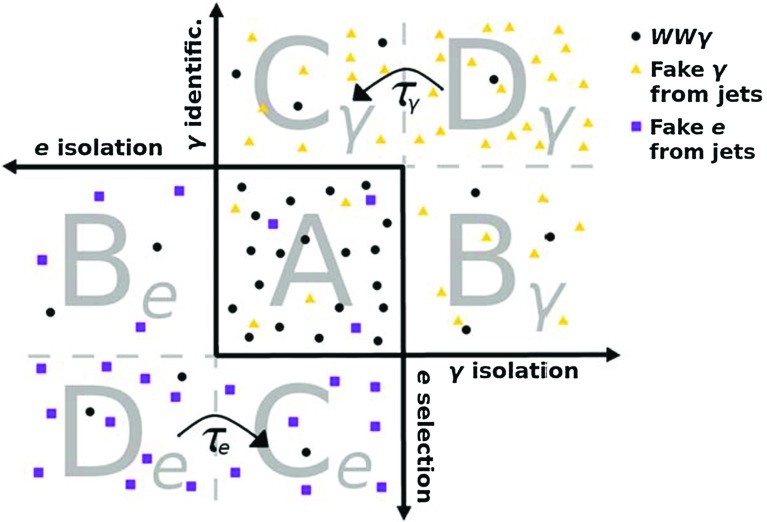
Schematic drawing of the combination of the two 2D sideband methods to estimate the background from events containing fake $\gamma$ (triangles) and fake *e* (squares) from jets. The $WW\gamma$ events are indicated with filled circles. The figure shows the signal region (region A) along with the six sideband regions. In regions C$_{\gamma }$ and D$_{\gamma }$ the requirement on the electron isolation stays unchanged as does the requirement on the photon isolation in regions C$_{e}$ and D$_{e}$. The factors $\tau _{\gamma }$ and $\tau _{e}$ that relate the event count in the isolated and non-isolated fake-object regions are also shown. The contributions of SM background processes to the different regions are omitted for simplicity

The production of *WW* and $t\bar{t}$ pairs in association with jets can mimic the signal if jets are misidentified as photons (fake $\gamma$ from jets). Jets can also be misidentified as muons (fake $\mu$ from jets) or electrons (fake *e* from jets) in which case $W\gamma$ + jets events can fulfil the signal selection criteria. The contribution from events containing fake $\mu$ from jets is determined from MC simulations and found to be very small. Events including fake $\gamma$ from jets or fake *e* from jets are removed from the MC simulation, as their contribution is estimated with data. These contributions are estimated by combining two two-dimensional (2D) sideband methods [69] (one per background component). A schematical drawing of the interplay between the methods is given in Fig. 2. It shows the three background-enriched sideband regions (B$_x$, C$_x$, D$_x$) per fake-object category *x* (with $x \in \{\gamma , e\}$) along with the signal region (A) that is common to the two fake-object categories. In the sideband regions, the contribution from signal and other SM processes containing prompt photons is accounted for using MC estimates. The method relies on the assumption that the definition of the sideband regions uses uncorrelated observables. Then, the ratio $\tau _{x}$ of the number of events in region C$_x$ ($N^{\text {fake}\,x}_{\text {C}_{x}}$) to the number of events in region D$_x$ ($N^{\text {fake}\,x}_{\text {D}_{x}}$) multiplied by the number of events in region B$_x$ ($N^{\text {fake}\,x}_{\text {B}_{x}}$) can be used to estimate the number of events containing fake objects of category *x* in region A ($N^{\text {fake}\,x}_{\text {A}}$). A possible correlation of the observables is accounted for by introducing the correlation factor $\rho _{x}$, which is set to one, representing no correlation, for the computation of the background contributions and varied to estimate the corresponding uncertainty.

The sideband regions B$_{\gamma }$, C$_{\gamma }$ and D$_{\gamma }$ are defined using the photon isolation, $E_{\text {T}}^{\text {iso,}\,\gamma }$, and a set of photon identification criteria related to the energy deposits in the first layer of the LAr calorimeter. The sideband regions B$_{e}$, C$_{e}$ and D$_{e}$ are defined using the electron isolation, $E_{\text {T}}^{\text {iso},\,e}$, and a set of electron–jet event selection criteria. The latter require the presence of at least one candidate electron and one jet with an absolute azimuthal separation of at least 0.7 in the event as well as $m_{\text {T}} \le {30\,\text {GeV}}$ and, if there is a second lepton in the event, the invariant mass of the lepton pair, $m_{\ell \ell }$, has to fulfil^2^ $|m_{\ell \ell }- m_{Z}| > {7\,\text {GeV}}$. The latter two criteria suppress the contribution of electrons originating from the decay of $W$ and $Z$ bosons, respectively.

As region A is common to the two fake-object categories, the estimation of the fake $\gamma$ and fake *e* from jets contributions in the signal region is performed simultaneously using a maximum likelihood approach. The likelihood function is the product of the Poisson probabilities of observing the expected number of events in the seven regions multiplied by Gaussian functions that incorporate the systematic uncertainties as nuisance parameters. This function has seven free parameters: the number of signal events in the signal region ($N^{e\nu \mu \nu \gamma }_{\text {obs}}$), the ratios $\tau _{\gamma }$ and $\tau _{e}$ as well as $N^{\text {fake}\,\gamma }_{\text {A}}$, $N^{\text {fake}\,e\phantom {\gamma }}_{\text {A}}$, $N^{\text {fake}\,\gamma }_{\text {C}_{\gamma }}$ and $N^{\text {fake}\,e\phantom {\gamma }}_{\text {C}_{e\phantom {\gamma }}}$. These parameters are determined by maximising the likelihood function that is constrained using the number of observed events in the seven regions defined by the method.

Apart from providing the contribution of fake $\gamma$ and fake *e* from jets in the signal region, the likelihood function also yields the most likely value of the number of signal events in the signal region: $N^{e\nu \mu \nu \gamma }_{\text {obs}} = 9.4 \pm 6.2$. This value is consistent with the difference between the number of observed events and the total background prediction given in Table 1. The former is used for the determination of the fiducial cross-section in Sect. 7. Several sources of systematic uncertainty are taken into account. Varying the correlation factor $\rho _{\gamma }$ ($\rho _{e}$) from one by its uncertainty $\Delta \rho _{\gamma }^{MC} = \pm \ 0.44$ ($\Delta \rho _{e}^{MC} = \pm \ 0.69$) as extracted from the MC simulation expectation, yields a relative uncertainty in $N^{e\nu \mu \nu \gamma }_{\text {obs}}$ of 10% (0.4%). The uncertainty in the number of events from SM processes in the sideband regions that are estimated from simulation is accounted for by varying the event yield by its total uncertainty and contributes 6% to the total uncertainty in $N^{e\nu \mu \nu \gamma }_{\text {obs}}$. The uncertainty in estimating the number of signal events in the sideband regions contributes less than 1% to the total uncertainty. The dominant uncertainty in $N^{e\nu \mu \nu \gamma }_{\text {obs}}$ originates from the limited number of data events and contributes a relative uncertainty of 60%.

**Fig. 3:**
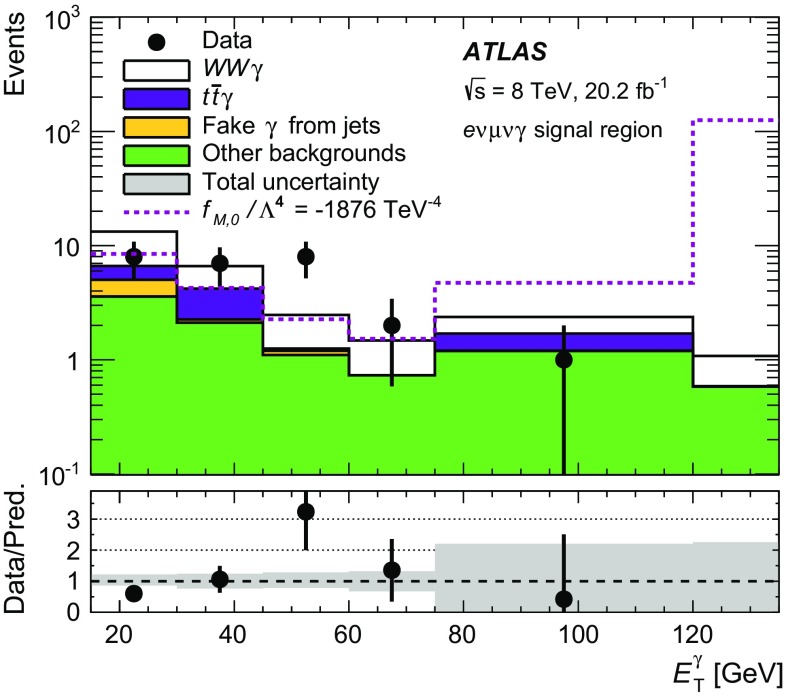
Observed and expected transverse energy distribution of the photon with the highest $E_{\text {T}}$ in the $e\nu \mu \nu \gamma$ signal region. The data are shown together with the predicted signal and backgrounds. Also indicated is the expected event yield for a reference model describing aQGCs with $f_{M,0}/\Lambda ^{4} = -1876\,\text {TeV}^{-4}$ (see Sect. 8). The last bin contains all overflow events. The lower panel shows the ratio of the observed number of events to the sum of expected signal and background events as well as the corresponding uncertainties

Figure 3 shows the transverse energy distribution of the photon with the highest $E_{\text {T}}$ in the signal region. The data are shown together with the expected signal from the MC prediction and the results from the background estimation. Also shown is the predicted event yield for a reference point in the parameter space of aQGCs discussed in Sect. 8. The lower panel of the figure shows the ratio of the number of observed events to the sum of the expected signal and background events.

## Analysis of semileptonic final states

In the semileptonic analysis, $WV \gamma$ production with one leptonically decaying $W$ boson and one hadronically decaying $W$ or $Z$ boson is studied. The event selection requires one lepton, at least two jets, at least one photon, and missing transverse momentum. The analysis is performed separately in the electron and the muon channels. The transverse momentum of the reconstructed electron or muon is required to be larger than 25 $\text {GeV}$. Events containing additional reconstructed electrons or muons with $p_{\text {T}} > {7\,\text {GeV}}$ are discarded. Photons are required to have $E_{\text {T}} > {15\,\text {GeV}}$. Jets are required to have $p_{\text {T}} > {25\,\text {GeV}}$ and to be within the volume of the tracking detector, $|\eta | < 2.5$, to ensure that jets originating from heavy-flavour quarks can be identified. In addition, the two jets with the highest transverse momenta are required to be close together with $|\Delta \eta _{jj}| < 1.2$ and $\Delta R_{jj} < 3.0$ to reject backgrounds from $W\gamma$ + jets events. The missing transverse momentum and the transverse mass of the event are both required to exceed 30 $\text {GeV}$. In events containing electrons, the invariant mass of the electron–photon pair is required to differ from the value of the $Z$ boson mass by at least 10 $\text {GeV}$ to suppress backgrounds from events containing leptonically decaying $Z$ bosons. To reduce background contributions from processes including top quarks, mainly $t\bar{t}\gamma$, events containing jets that are identified as originating from the decay of a *b*-hadron are rejected. The *b*-jet identification is performed using the MV1 algorithm [71] based on an artificial neural network with an efficiency of 85% and a light-quark-jet and gluon-jet misidentification rate of 10%. Finally, the invariant mass of the two jets with the highest transverse momenta in the event is required to be close to the mass of the decaying $W$ or $Z$ boson, i.e. ${70\,\text {GeV}}< m_{jj} < {100\,\text {GeV}}$. These selection requirements are optimised to yield the best sensitivity to the signal and define the signal region. The expected number of signal events is $14 \pm 2$ ($18 \pm 2$) in the electron (muon) channel, as computed with the VBFNLO program and corrected for acceptance and efficiency effects (described in Sect. 7 along with the corresponding uncertainties). A total of 490 (599) events are observed in the electron (muon) channel.

**Table 2:** Expected and observed event yields in the signal region of the electron and muon channels of the semileptonic analysis. For each background process the corresponding estimation method is stated. The uncertainties of the $W\gamma$ + jets, fake $\gamma$ from jets and fake $\ell$ from jets are solely the statistical uncertainties from data. The uncertainties of the $t\bar{t}\gamma$, fake $\gamma$ from *e*, $Z\gamma$ + jets and $WV \gamma \rightarrow \tau \nu jj \gamma$ backgrounds correspond to the sum in quadrature of the statistical uncertainty of the MC simulation and the uncertainties of the theoretical prediction. The uncertainty in the total background estimate is symmetrised and contains the statistical uncertainty of the data, the uncertainties of the theoretical prediction, and experimental uncertainties. The expected signals are computed with the VBFNLO program and corrected for acceptance and efficiency

Process	Electron channel	Muon channel	Estimation method
$W\gamma$ + jets	324 ± 11	407 ± 11	Simultaneous fit
Fake $\gamma$ from jets	82 ± 7	117 ± 9	Simultaneous fit
Fake $\ell$ from jets	57 ± 6	27 ± 5	Simultaneous fit
$t\bar{t}\gamma$	35 ± 6	46 ± 7	MC simulation
Fake $\gamma$ from *e*	33 ± 12	3 ± 1	Corrected simulation
$Z\gamma$ + jets	19 ± 4	20 ± 3	MC simulation
$WV \gamma$ ($\tau$ contribution)	<1	<1	MC simulation
Total background	552 ± 38	621 ± 31	Sum of components
Expected signal	14 ± 2	18 ± 2	Corrected VBFNLO
Data	490	599	Measurement

The background processes of the semileptonic analysis are listed in Table 2. The dominant contribution arises from $W\gamma$ + jets production, as it has the same final state as the signal. The contribution from $t\bar{t}\gamma$, $Z\gamma$ + jets as well as from $WV \gamma$ processes containing $\tau$ leptons ($WV \gamma \rightarrow \tau \nu jj \gamma$) processes, is estimated using MC simulation. The uncertainties in these background contributions given in Table 2 solely include statistical uncertainties and the uncertainties of the theoretical prediction, that are of the same size. The relative uncertainties of the theoretical predictions range from 4 to 22% [6, 40]; the uncertainties associated with the computation of the $WV \gamma$ process are described in Sect. 7. The experimental uncertainties are only included in the uncertainty of the total background estimation in Table 2, as they are correlated for the individual background components.

Events containing misidentified objects constitute an important source of background in this analysis as well. When electrons are misidentified as photons (fake $\gamma$ from *e*), $Z \rightarrow ee$ production in association with jets and $t\bar{t}$ events can mimic the signal. As in the fully leptonic analysis, this background is estimated using MC simulation which is corrected to match the misidentification rate measured in data. The uncertainty of this correction is small compared to the statistical uncertainty and the uncertainties from the theoretical calculation. The latter uncertainty is estimated to be 5% for the $Z \rightarrow ee$ and the $t\bar{t}$ processes in agreement with the corresponding measurements [72, 73]. Mainly events from $W \ +$ jets production contribute as background when a jet is misidentified as a photon (fake $\gamma$ from jets). In events containing jets misidentified as leptons (fake $\ell$ from jets) predominantly $\gamma \ +$ jets production constitues a background. Events containing fake $\gamma$ from jets or fake $\ell$ from jets are removed from the MC simulation, as their contribution is estimated with data.

A simultaneous fit is used to estimate the background contributions from $W\gamma$ + jets production and from events containing fake $\gamma$ from jets and fake $\ell$ from jets (the fake *e* from jets component also includes the small contribution from fake *e* from $\gamma$). The simultaneous fit consists of three components: a binned extended maximum-likelihood fit of the invariant dijet mass distribution to constrain the $W\gamma$ + jets contribution, a binned extended maximum-likelihood fit of the $E_{\text {T}}^{\text {miss}}$ distribution to constrain the fake $\ell$ backgrounds and a two-dimensional sideband method to constrain the contribution from fake $\gamma$ from jets. The free parameters of the simultaneous fit are the normalisation of the $W\gamma$ + jets background, the normalisation of the processes containing fake $\ell$ from jets and the normalisation of the processes containing fake $\gamma$ from jets. The normalisation of all other background components is fixed. The fit is performed separately in the electron and muon channels of the analysis. For all three estimation methods the signal region with ${70\,\text {GeV}}< m_{jj} < {100\,\text {GeV}}$ is excluded such that the overall signal contribution to the fiducial region used for the background estimation is negligible. Therefore, the signal contribution in all regions used in the fit is neglected and the result is independent of the signal modelling. The $m_{jj}$ distribution is fitted in the range 10–70 and 100–505 $\text {GeV}$; the $E_{\text {T}}^{\text {miss}}$ distribution is fitted in the range 0–300 $\text {GeV}$. No minimum $E_{\text {T}}^{\text {miss}}$ requirement is imposed in the fit of the $E_{\text {T}}^{\text {miss}}$ distribution, in order to increase the sensitivity to fake $\ell$ from jets, as these events are expected to have low missing transverse momentum. Apart from neglecting the signal contribution, the two-dimensional sideband method is performed as for the fake photons from jets in the fully leptonic analysis.

The extended likelihood fits employ shape templates for the $m_{jj}$ and $E_{\text {T}}^{\text {miss}}$ distributions of the different background components. The shape templates for all backgrounds are derived from simulation apart from the ones associated with fake $\ell$ from jets and fake $\gamma$ from jets. The latter shape templates are obtained from data events selected similarly to the fit regions with some requirements modified as follows to enhance the contribution from the respective fake object. To estimate the shape template for fake *e* from jets, the requirement on $E_{\text {T}}^{\text {miss}}$ is removed and the requirements on the electron identification and isolation are modified. To this end, the requirements on the calorimeter-based isolation and the origin of the electron track are removed and the track-based isolation requirement is inverted. To estimate the shape template for fake $\mu$ from jets, the requirement on $E_{\text {T}}^{\text {miss}}$ is removed and the requirements on the muon isolation and the origin of the track are inverted. To estimate the shape template for fake $\gamma$ from jets, the requirement on the photon isolation is removed and at least one of the photon identification criteria based on the energy deposits in the first layer of the LAr calorimeter must not be satisfied. The $m_{jj}$ shape templates are also employed to extrapolate the background estimation results of the different background components to the signal region.

**Fig. 4:**
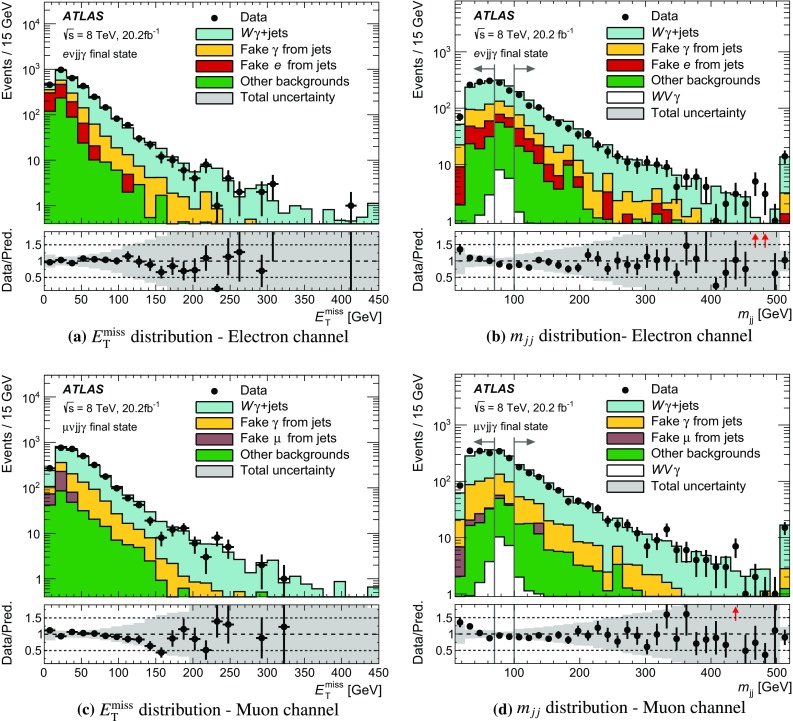
Missing transverse momentum and dijet invariant mass distributions of the electron (upper row) and the muon channels (lower row) of the semileptonic analysis. The different background components are shown together with the data. The signal region (${70\,\text {GeV}}< m_{jj} < {100\,\text {GeV}}$) is excluded in (**a**) and (**c**) as well as in the simultaneous fit as indicated by the arrows in (**b**) and (**d**). The last bin of each figure contains the event overflow. The lower panels show the ratio of the observed number of events to the predicted background as well as the corresponding uncertainties. The red arrows indicate entries that are outside the *y*-axis range

Figure 4 shows the results of the simultaneous fit, in the upper panel for the electron channel and in the lower panel for the muon channel. In Fig. 4a, c the resulting $E_{\text {T}}^{\text {miss}}$ distributions are presented; the events are selected using the criteria for the signal region, but the requirement on $E_{\text {T}}^{\text {miss}}$ is removed and the requirement on $m_{jj}$ is inverted. The lower panels of the figures show the ratio of the observed number of events to the expected number of events, which agrees with unity within uncertainties. In Fig. 4b, d the resulting $m_{jj}$ distributions are shown. All signal selection requirements apart from the $m_{jj}$ requirement are imposed. The distribution observed in data is underestimated by the background estimation in both channels at low $m_{jj}$ values but agrees within uncertainties. As a cross check, an alternative shape template for the $W\gamma$ + jets background is obtained from simulated events generated with SHERPA. While the resulting background estimate shows better agreement with the data at low values of $m_{jj}$, no significant impact on the background estimate in the signal region is found. The event yields of the $W\gamma$ + jets, fake $\gamma$ from jets and fake $\ell$ from jets events in the signal region are given in Table 2. The uncertainties in these components in Table 2 correspond solely to the statistical uncertainty from data.

The uncertainty in the total number of background events has several sources. The uncertainty associated with the shape templates is estimated by performing 10,000 pseudo experiments that use alternative shape templates obtained from sampling the nominal ones bin-wise using a Gaussian distribution. The width of the Gaussian distribution corresponds to the statistical uncertainty of the shape templates determined from data, or to the statistical uncertainty of the MC simulation and the uncertainties from the theoretical calculation if they are determined from simulation. The shape templates are varied simultaneously and yield an uncertainty in the total background of 5% (4%) in the electron (muon) channel. The experimental uncertainties are the uncertainties due to reconstruction and identification efficiencies of the objects [50, 52, 54, 74, 75] including energy scale and energy resolution uncertainties [52, 55, 60, 67, 68] as well as uncertainties arising from the simulation of the event pile-up [61]. These uncertainties are estimated for all background components simultaneously and amount to a total of 4 (3%) in the electron (muon) channel. They are dominated by the uncertainty in the jet energy scale. The uncertainty related to the choice of fit boundaries for the extended maximum-likelihood fits is estimated by varying these boundaries. The lower $m_{jj}$ ($E_{\text {T}}^{\text {miss}}$) boundary is set to 25 (15 $\text {GeV}$) and the upper boundary is set to 490 or 520 $\text {GeV}$ (285 or 315 $\text {GeV}$) independently. The uncertainty introduced by the choice of binning for the distributions used for the extended maximum-likelihood fits is estimated by varying the bin sizes by a factor of two. The uncertainty due to the possible correlation of the selection criteria defining the sideband regions of the 2D sideband method is estimated by changing the value of the correlation factor $\rho$ from one by its uncertainty $\Delta \rho _{e\nu jj \gamma }^{MC} = \pm \ 0.38$ ($\Delta \rho _{\mu \nu jj \gamma }^{MC} = \pm \ 0.23$) as extracted from the MC simulation expectation. The uncertainty associated with any of these fit parameter variations is less than 1% in each channel of the analysis. The statistical uncertainty in the expected total number of background events corresponds to 2.6 (2.5%) in the electron (muon) channel.

**Fig. 5:**
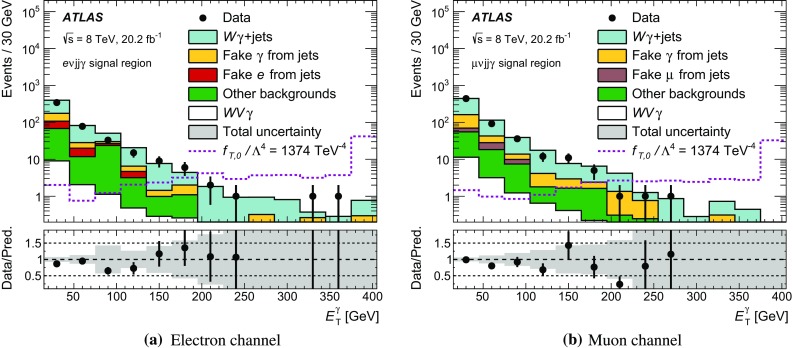
Observed and expected transverse energy distributions of the photon with the highest $E_{\text {T}}$ in the signal region in the **a** electron and **b** muon channels of the semileptonic analysis. The data are shown together with the predicted signal and backgrounds. Also indicated is the expected event yield for a reference model describing aQGCs with $f_{T,0}/\Lambda ^{4} = 1374\,\text {TeV}^{-4}$ (see Sect. 8). The last bin of each figure contains all overflow events. The lower panels show the ratio of the observed number of events to the sum of expected signal and background events as well as the corresponding uncertainties

Figure 5 shows the transverse energy distributions of the photon with the highest $E_{\text {T}}$ in the signal region in the electron and the muon channels. The data are shown together with the estimated background contributions and the expected signal yield. The expected distribution for a reference point in the parameter space of aQGCs (see Sect. 8) is also indicated. The lower panels of the two figures show the ratios of the number of observed events to the sum of expected signal and background events.

## Production cross-section

The cross-section for $WV \gamma$ production is determined in fiducial regions close to the signal regions defined in Sects. 5 and 6. While the signal region definition is based on reconstructed objects, the definition of the fiducial region is based on particle-level MC generator information. The latter corresponds to the MC simulation including the parton shower, hadronisation and underlying event, as opposed to the parton level, which does not account for these effects and solely includes the hard-scattering process of the event.

At particle level, jets are reconstructed from all stable particles (traveling at least 10 mm before decaying) in the final state, except for muons and neutrinos, using the anti-$k_{t}$ algorithm with $R = 0.4$. The identification of *b*-jets at particle level is based on a matching of the jets to *b*-hadrons within a cone of size $\Delta R = 0.3$ around the jet axis. The final-state radiation of photons from leptons is accounted for by adding the four-momenta of photons that lie within a cone of size $\Delta R = 0.1$ around a lepton to the lepton four-momentum. The missing transverse momentum of a particle-level event is obtained from the momenta of the neutrinos in the final state.

The selection criteria defining the fiducial region are summarised in Table 3. They differ from the criteria defining the signal region only for the requirements on the pseudorapidity range and the isolation of the objects. Leptons are required to fulfil $|\eta | < 2.5$ and photons $|\eta | < 2.37$. Thus, the transition region ($1.37< |\eta | < 1.52$) is included in the fiducial region and the $\eta$ requirements of the electrons and muons are unified. No isolation requirements are imposed on electrons or muons. The photon isolation requirement is based on the isolation fraction $\epsilon _{h}^{p}$. The latter is defined as the ratio of the transverse energy of the closest jet that lies within a cone of size $\Delta R = 0.4$ around the photon to the transverse energy of the photon. Photons are considered isolated when $\epsilon _{h}^{p} < 0.5$.

**Table 3:** Definition of the fiducial regions of the fully leptonic and semileptonic $WV \gamma$ analyses. The objects are defined at particle level and the $\Delta R$ requirements are employed in the overlap removal. The latter is implemented differently for electrons and muons. For electron–jet pairs failing the $\Delta R(\text {jet}, \ell )$ requirement, the jet candidate is discarded and for muon–jet pairs failing the requirement, the muon candidate is discarded

	Fiducial Requirements	
	$e\nu \mu \nu \gamma$	$\ell \nu jj\gamma$
Leptons	1 electron and 1 muon	1 electron or 1 muon
	$p_{\text {T}} > {20\,\text {GeV}}$	$p_{\text {T}} > {25\,\text {GeV}}$
	No 3rd lepton ($p_{\text {T}} > {7\,\text {GeV}}$)	No 2nd lepton ($p_{\text {T}} > {7\,\text {GeV}}$)
	$|\eta | < 2.5$	$|\eta | < 2.5$
	Opposite charge leptons	
	$$\Delta R(\ell , \ell ^{\prime }) > 0.1$$	
Photon	$\ge$1 isolated photon	
	$$E_{\text {T}} > {15\,\text {GeV}}$$	
	Isolation fraction $\epsilon _{h}^{p} < 0.5$	
	$$|\eta | < 2.37$$	
	$$\Delta R(\ell , \gamma ) > 0.5$$	
Jets	$N_{\text {jets}} = 0$	$N_{\text {jets}} \ge 2$ and $N_{\text {b-jets}} = 0$
	$p_{\text {T}} > {25\,\text {GeV}}$	$p_{\text {T}} > {25\,\text {GeV}}$
	$|y| < 4.4$	$|\eta | < 2.5$
		$$|\Delta \eta _{jj}| < 1.2$$
		$$\Delta R_{jj} < 3.0$$
		$${70\,\text {GeV}}< m_{jj} < {100\,\text {GeV}}$$
	$\Delta R(\text {jet}, \gamma ) > 0.5$	$\Delta R(\text {jet}, \gamma ) > 0.5$
	$\Delta R(\text {jet}, \ell ) > 0.3$	$\Delta R(\text {jet}, \ell ) > 0.3$
$W$ boson	$E_{\text {T,\,rel}}^{\text {miss}} > {15\,\text {GeV}}$	$E_{\text {T}}^{\text {miss}} > {30\,\text {GeV}}$
	$m_{e\mu }> {50\,\text {GeV}}$	$m_{\text {T}} > {30\,\text {GeV}}$

### Cross-section predictions

The cross-section predictions are computed at NLO in $\alpha _{\text {S}}$ using the VBFNLO program. The computations are performed at parton level, while the measurement is performed at particle level. Therefore, the cross-section predictions are corrected to particle level by multiplying them by the parton-to-particle-level correction factors ($C^{\text {p2p}}$). Each correction factor is defined as the number of signal events that satisfy the selection criteria for the fiducial region defined at particle level divided by the number of signal events that satisfy the selection criteria for the fiducial region defined at parton level. These factors are evaluated using the SHERPA signal simulation and amount to $1.10 \pm 0.01$, $0.64 \pm 0.01$ and $0.57 \pm 0.02$ for the $e\nu \mu \nu \gamma$, $e\nu jj\gamma$ and $\mu \nu jj\gamma$ final states, respectively. The main difference between these corrections for the fully leptonic and the semileptonic final states arises from the fundamentally different requirements on the presence of jets and partons in the events. The difference between the electron and muon channels in the semileptonic analysis arises from different overlap removal algorithms employed for electrons and muons; while jet candidates are discarded when they are close to electrons, muon candidates are discarded when they are reconstructed close to a jet, to remove contributions from heavy-flavour quark decays. The uncertainties of the parton-to-particle-level correction factors include the statistical uncertainty of the SHERPA sample and a systematic component evaluated as the difference between the corrections estimated with the SHERPA and the MadGraph signal samples. The latter uncertainty accounts for differences in the parton shower modelling and the description of the underlying event between the two generators. The expected cross-section at particle level for the different final states and for the average of the electron and muon channels of the semileptonic analysis ($\ell \nu jj\gamma$) are summarised in Table 4. The expected cross-sections for the fully leptonic and semileptonic final states are of similar size despite the larger hadronic branching fraction of the $W$ and $Z$ bosons, as the selection criteria for the fiducial regions in the semileptonic analysis are more restrictive. The uncertainty in the expected cross-section is about 5% for all final states. This value accounts for the uncertainty associated with $C^{\text {p2p}}$, the numerical accuracy of the calculation, variations of the renormalisation and factorisation scales ($\mu _{\text {R}}$ and $\mu _{\text {F}}$) by a factor of two (varied independently with the constraint $0.5 \le \mu _{\text {F}}/\mu _{\text {R}} \le 2$), uncertainties due to the choice of PDF set and value of the strong coupling constant $\alpha _{\text {S}}$ as well as uncertainties due to the choice of isolation fraction requirement evaluated by changing the criterion by $\pm \,0.25$. No additional uncertainty related to the scale introduced by restricting the jet multiplicity in the fully leptonic analysis is taken into account. This uncertainty has been shown to be of the same order as the already included scale uncertainty by studying $W$-boson pair production [76]. Accordingly, no additional uncertainty is considered here as the experimental uncertainties are comparatively large and its inclusion would not change the results of this analysis.

### Cross-section determination

The observed production cross-section is determined from the number of signal events in the signal region, $N_{\text {obs}}$, and the integrated luminosity of the data set, $L_{\text {int}}$, according to $\sigma _{\text {fid}} = N_{\text {obs}}/(\epsilon L_{\text {int}}),$ where the correction factor, $\epsilon$, accounts for the different geometrical acceptance and selection efficiencies of the signal region defined using reconstructed objects and the fiducial region defined at particle level. The correction factor is evaluated using the SHERPA signal simulation and amounts to $0.30 \pm 0.02$ for the $e\nu \mu \nu \gamma$ final state and to $0.28 \pm 0.02$ ($0.40 \pm 0.03$) for the electron (muon) channel of the semileptonic analysis. The larger ranges in pseudorapidity of the leptons and photons in the fiducial region compared to the signal region contribute about 11% to $\epsilon$. The uncertainties of $\epsilon$ include the experimental uncertainties associated with the signal, a statistical component, and a systematic component evaluated as the difference between the corrections estimated with the SHERPA and the MadGraph signal sample to account for differences in the parton shower modelling and the description of the underlying event. The latter yields the largest contribution to the total uncertainty with the second largest contribution being the uncertainty associated with the jet energy scale.

For the fully leptonic analysis, the fiducial cross-section computed using $N^{e\nu \mu \nu \gamma }_{\text {obs}}$ from Sect. 5 is

$$\begin{aligned} \sigma _{\text {fid}}^{e\nu \mu \nu \gamma } = 1.5 \pm 0.9 (\text {stat.}) \pm 0.5 (\text {syst.})\,\text {fb}, \end{aligned}$$

where the uncertainties are symmetrised and the luminosity uncertainty is included as part of the systematic uncertainty. The observed (expected) significance of this cross-section is determined by evaluating the *p* value of the background-only hypothesis at 95% confidence level, CL, and corresponds to 1.4 $\sigma$ sigma (1.6 $\sigma$). The *p* value is calculated using a maximum likelihood ratio as the test statistic. This determination of the $e\nu \mu \nu \gamma$ production cross-section is in agreement with the theory prediction from Table 4 corresponding to 2.0 fb. The cross-section is not determined in the semileptonic final states due to its smaller significance.

Upper limits on the production cross-sections are computed for the $e\nu \mu \nu \gamma$, $e\nu jj\gamma$ and $\mu \nu jj\gamma$ final states and for the average cross-section per lepton flavour ($\ell \nu jj\gamma$) in the semileptonic final states. They are determined at 95% CL using the CL$_{\text {s}}$ technique [77]. For the combination of the semileptonic final states, the product of the likelihood functions of the $e\nu jj\gamma$ and $\mu \nu jj\gamma$ final states is used as the $\ell \nu jj\gamma$ likelihood function in the CL$_{\text {s}}$ method. The expected limits in the absence of a signal are computed using an Asimov data set [78], which provides an analytical approximation of the distribution of expected limits based on a $\chi ^2$-distribution of the test statistics. The observed and expected limits are listed in Table 4. The observed limits are between 1.8 and 4.1 times larger than the SM cross-section. The observed upper limit on the $\ell \nu jj\gamma$ production cross-section is the most stringent limit reported to date.

**Table 4:** Observed and expected cross-section upper limits at 95% CL for the different final states using the CL$_{\text {s}}$ method. The expected cross-section limits are computed assuming no signal is present. The last column shows the theory prediction for the signal cross-section ($\sigma _{\text {theo}}$) computed with the VBFNLO program and corrected to particle level. The $\ell \nu jj\gamma$ cross-section corresponds to the average cross-section per lepton flavour in the semileptonic analysis and all events of the $e\nu jj\gamma$ and $\mu \nu jj\gamma$ final states are employed for the determination of this limit

	Observed limit [fb]	Expected limit [fb]	$\sigma _{\text {theo}}$ [fb]
Fully leptonic $e\nu \mu \nu \gamma$	3.7	$2.1^{+0.9}_{-0.6}$	$2.0 \pm 0.1$
Semileptonic $$\left\{ \begin{array}{l} e\nu jj\gamma \\ \mu \nu jj\gamma \\ \ell \nu jj\gamma \end{array} \right. $$	$$\begin{array}{l} 10 \\ 8 \\ 6 \end{array}$$	$$\begin{array}{l} 16^{+6}_{-4} \\ 10^{+4}_{-3} \\ 8.4^{+3.4}_{-2.4}\end{array}$$	$$\begin{array}{l} 2.4 \pm 0.1 \\ 2.2 \pm 0.1 \\ 2.3 \pm 0.1 \end{array}$$

## Search for new physics beyond the Standard Model

In addition to the results derived in the previous chapter, exclusion limits on the production cross-section and conficence intervals on aQGCs are derived in a fiducial region optimised for a search for new physics beyond the SM. This fiducial region differs from the fiducial region defined in Sect. 7 by an increased photon $E_{\text {T}}$ requirement.

The aQGCs are introduced by extending the SM Lagrangian density function ($\mathcal {L}_{\text {SM}}$) with terms containing operators ($\mathcal {O}_{x}$) of energy-dimension eight as this is the lowest dimension that describes quartic gauge boson couplings without exhibiting triple gauge-boson vertices [79]. The operators consist of different combinations of the SM fields and their coefficients are written as the ratio of a coupling parameter ($f_{x}$) to the fourth power of the energy scale ($\Lambda$) at which the new physics beyond the SM would occur. Thus, the effective Lagrangian density ($\mathcal {L}_{\text {eff}}$) for $WV \gamma$ production can be written as:

3 $$\begin{aligned} \mathcal {L}_{\text {eff}} = \mathcal {L}_{\text {SM}} + \sum _{j=0}^{7} \frac{f_{M,j}}{\Lambda ^{4}}\mathcal {O}_{M,j} + \sum _{j=0,1,2,5,6,7} \frac{f_{T,j}}{\Lambda ^{4}}\mathcal {O}_{T,j}, \end{aligned}$$

as there are 14 different operators that describe anomalous $WWZ\gamma$ and $WW\gamma \gamma$ couplings. The indices *T* and *M* of the coupling parameter indicate two different classes of aQGC operators: operators containing only field strength tensors (*T*) and operators containing field strength tensors and the covariant derivative of the Higgs field (*M*). The SM prediction of each of the coupling parameters is zero. The reference models in Figures 3 and 5 depict values that are excluded by previous analyses.

The effective field theory is not a complete model and violates unitarity at sufficiently high energy scales. This violation can be avoided by multiplying the coupling parameters with a dipole form factor of the form:

4 $$\begin{aligned} \frac{1}{(1 + \hat{s}/\Lambda ^2_{\text {FF}})^{2}}, \end{aligned}$$

as described in Ref. [80]. Here, $\hat{s}$ corresponds to the squared invariant mass of the produced bosons and $\Lambda _{\text {FF}}$ is the energy scale of the form factor. The latter corresponds to the energy regime above which the contributions of the anomalous couplings are largely suppressed. For triboson processes there is no theoretical algorithm to compute the appropriate value for $\Lambda _{\text {FF}}$ to avoid unitarity violation. Therefore, the confidence intervals in this analysis are derived using three different values of $\Lambda _{\text {FF}}$: 0.5, 1 $\text {TeV}$ and infinity. The latter corresponds to the non-unitarised case, which is evaluated to allow for the comparison with other analyses.

For the determination of the confidence intervals, only one coupling parameter is varied at a time and all others are set to zero. The expected number of events as a function of the varied parameter is described by a quadratic function and the predictions of the VBFNLO program corrected to particle level are used for the determination of this function. Confidence intervals at 95% CL are computed using a maximum profile-likelihood ratio test statistic as done in Ref. [69].

**Table 5:** Numbers of observed events ($N_{\text {obs}}$) and predicted background events ($N_{\text {bg}}$) for the different final states with the respective photon $E_{\text {T}}$ threshold optimised for maximal aQGC sensitivity. Also given are the correction factors $\epsilon$ to correct from reconstruction level to particle level and $C^{\text {p2p}}$ to correct from parton level to particle level

	$E_{\text {T}} ^{\gamma }$ threshold [$\text {GeV}$]	$N_{\text {obs}}$	$N_{\text {bg}}$	$\epsilon$	$C^{\text {p2p}}$
$e\nu \mu \nu \gamma$	120	0	$0.1^{+\,0.2}_{-\,0.1}$	0.3 ± 0.1	1.1 ± 0.1
$e\nu jj\gamma$	200	4	$6\pm 6$	0.4 ± 0.1	0.6 ± 0.2
$\mu \nu jj\gamma$	200	3	$4^{+\,12}_{-\,4}$	0.4 ± 0.1	0.6 ± 0.1

The aQGCs would modify $WV \gamma$ production at high values of $\hat{s}$ such that the sensitivity to aQGCs can be improved by raising the threshold of the transverse energy of the photon. As the event count in the signal region decreases with an increasing $E_{\text {T}} ^{\gamma }$ threshold, the expected background contribution from the other processes is extrapolated from the results obtained in Sects. 5 and 6 with $E_{\text {T}} ^{\gamma } > {15\,\text {GeV}}$. To this end, the $E_{\text {T}} ^{\gamma }$ distribution of the total background prediction is fitted using an exponential function (the sum of two exponential functions) in the fully leptonic (semileptonic) analysis and the total background yield is derived from the fit. The optimal value of the $E_{\text {T}} ^{\gamma }$ threshold is determined by varying the threshold, computing the expected confidence intervals for all 14 parameters and choosing the threshold that yields the smallest expected intervals for each final state individually. This optimisation yields the best sensitivity for the requirement $E_{\text {T}} ^{\gamma } > {120\,\text {GeV}}$ in the fully leptonic analysis and for $E_{\text {T}} ^{\gamma } > {200\,\text {GeV}}$ in both channels of the semileptonic analysis.

The number of observed events and the expected number of background events above the optimised $E_{\text {T}} ^{\gamma }$ threshold are given in Table 5. The uncertainty in the background estimation includes the uncertainty in the original background estimation and an additional uncertainty due to the extrapolation procedure, which is dominant. The latter is evaluated by varying the fit range as well as evaluating the impact of the uncertainty of the fit parameters on the background estimation. Due to the higher $E_{\text {T}} ^{\gamma }$ threshold, the factors $\epsilon$ and $C^{\text {p2p}}$ are recomputed using the SM signal samples and are also listed in Table 5. As an additional source of systematic uncertainty, $\epsilon$ and $C^\mathrm {p2p}$ are evaluated using the aQGC simulated samples, and their maximal deviations from the SM predictions are considered to account for their dependence on the aQGC coupling. This uncertainty is the dominant one for $C^{\text {p2p}}$ in the fully leptonic analysis.

The upper limits on the $WV \gamma$ production cross-section in the high-$E_{\text {T}}$ photon fiducial region are computed using the CL$_{\text {s}}$ formalism at 95% CL. The results are given in Table 6 together with limits expected in absence of $WV \gamma$ production. In addition, the theory prediction for the SM signal cross-section computed with the VBFNLO program and corrected to particle level is reported. The cross-section uncertainties are evaluated as described in Sect. 7.1 and range up to 22%.

**Table 6:** Observed and expected cross-section upper limits at 95% CL using the CL$_{\text {s}}$ method for the different final states with the photon $E_{\text {T}}$ threshold optimised for maximal aQGC sensitivity. The expected cross-section limits are computed assuming the absence of $WV \gamma$ production. The last column shows the theory prediction for the SM signal cross-section computed with the VBFNLO program and corrected to particle level. The $\ell \nu jj\gamma$ cross-section corresponds to the average cross-section per lepton flavour in the semileptonic analysis and all events of the $e\nu jj\gamma$ and $\mu \nu jj\gamma$ final states are employed for the determination of this limit

	$E_{\text {T}} ^{\gamma }$ threshold	Observed	Expected	SM Prediction
	[$\text {GeV}$]	limit [fb]	limit [fb]	$\sigma _{\text {theo}}$ [fb]
Fully leptonic $e\nu \mu \nu \gamma$	120	0.3	$0.3^{+0.3}_{-0.1}$	0.076 ± 0.004
Semileptonic $$\left\{ \begin{array}{l}e\nu jj\gamma \\ \mu \nu jj\gamma \\ \ell \nu jj\gamma \end{array} \right. $$	$$\begin{array}{l} 200 \\ 200 \\ 200 \end{array}$$	$$\begin{array}{l} 1.3 \\ 1.1 \\ 0.9 \end{array}$$	$$\begin{array}{l} 1.3^{+0.5}_{-0.3}\\ 1.1^{+0.5}_{-0.3}\\ 0.9^{+0.3}_{-0.2}\end{array}$$	$$\begin{array}{l} 0.057 \pm 0.013 \\ 0.051 \pm 0.011 \\ 0.054 \pm 0.009 \end{array}$$

**Fig. 6:**
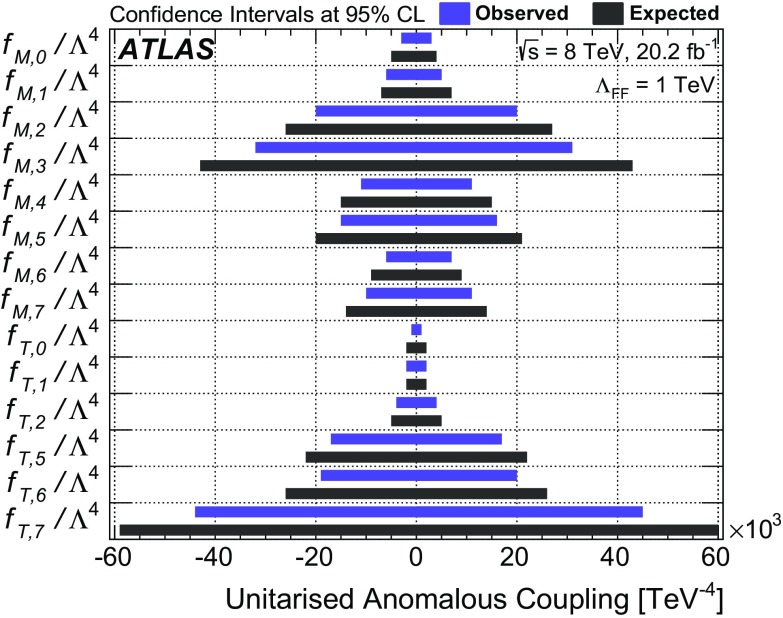
Observed and expected confidence intervals at 95% CL on the different anomalous quartic gauge couplings for the combined $WV \gamma$ analysis. The couplings are unitarised using a dipole form factor with a form factor energy scale of $\Lambda _{\text {FF}} = {1\,\text {TeV}}$

For the computation of the confidence intervals, the $e\nu \mu \nu \gamma$, $e\nu jj\gamma$ and $\mu \nu jj\gamma$ final states are combined. The test statistic is computed from the product of the likelihood functions of the individual final states. This combination improves the confidence intervals by up to 11% compared to the results obtained with the $e\nu \mu \nu \gamma$ final state only. The results are given in Table 7. In Fig. 6 the expected and observed confidence intervals using the form factor scale $\Lambda _{\text {FF}} = 1\,\text {TeV}$ are shown. The non-unitarised couplings have also been studied by other analyses (e.g. [5–13, 17]) and found to be consistent with the SM prediction of zero as confirmed by this analysis.

**Table 7:** Observed and expected confidence intervals at 95% CL on the different anomalous quartic gauge couplings for the combined $WV \gamma$ analysis for three different values of the form factor scale $\Lambda _{\text {FF}}$

Coupling	$\Lambda _{\text {FF}} = \infty$		$\Lambda _{\text {FF}} = {1\,\text {TeV}}$		$\Lambda _{\text {FF}} = {0.5\,\text {TeV}}$	
	Observed [$10^{3}$ $\text {TeV}$ $^{-4}$]	Expected [$10^{3}$ $\text {TeV}$ $^{-4}$]	Observed [$10^{4}$ $\text {TeV}$ $^{-4}$]	Expected [$10^{4}$ $\text {TeV}$ $^{-4}$]	Observed [$10^{4}$ $\text {TeV}$ $^{-4}$]	Expected [$10^{4}$ $\text {TeV}$ $^{-4}$]
$f_{M,0}/\Lambda ^{4}$	[−0.3, 0.3]	[−0.4, 0.4]	[−0.3, 0.3]	[−0.4, 0.5]	[−1.7, 1.8]	[−2.3, 2.4]
$f_{M,1}/\Lambda ^{4}$	[−0.5, 0.5]	[−0.8, 0.7]	[−0.6, 0.5]	[−0.7, 0.7]	[−2.9, 2.6]	[−4.0, 3.7]
$f_{M,2}/\Lambda ^{4}$	[−1.8, 1.8]	[−2.4, 2.5]	[−2.0, 2.0]	[−2.6, 2.7]	[−9.9, 10]	[−14, 14]
$f_{M,3}/\Lambda ^{4}$	[−3.1, 3.0]	[−4.2, 4.3]	[−3.2, 3.1]	[−4.3, 4.3]	[−17, 16]	[−23, 23]
$f_{M,4}/\Lambda ^{4}$	[−1.1, 1.1]	[−1.5, 1.6]	[−1.1, 1.1]	[−1.5, 1.5]	[−5.7, 6.2]	[−7.9, 8.4]
$f_{M,5}/\Lambda ^{4}$	[−1.7, 1.7]	[−2.3, 2.3]	[−1.5, 1.6]	[−2.0, 2.1]	[−8.0, 9.0]	[−11, 12]
$f_{M,6}/\Lambda ^{4}$	[−0.6, 0.6]	[−0.9, 0.9]	[−0.6, 0.7]	[−0.9, 0.9]	[−3.3, 3.5]	[−4.7, 4.9]
$f_{M,7}/\Lambda ^{4}$	[−1.1, 1.1]	[−1.5, 1.5]	[−1.0, 1.1]	[−1.4, 1.4]	[−5.2, 5.9]	[−7.5, 8.0]
$f_{T,0}/\Lambda ^{4}$	[−0.1, 0.1]	[−0.2, 0.2]	[−0.1, 0.1]	[−0.2, 0.2]	[−0.9, 0.8]	[−1.1, 1.1]
$f_{T,1}/\Lambda ^{4}$	[−0.2, 0.2]	[−0.2, 0.2]	[−0.2, 0.2]	[−0.2, 0.2]	[−0.9, 0.9]	[−1.2, 1.2]
$f_{T,2}/\Lambda ^{4}$	[−0.4, 0.4]	[−0.5, 0.5]	[−0.4, 0.4]	[−0.5, 0.5]	[−1.9, 2.0]	[−2.7, 2.7]
$f_{T,5}/\Lambda ^{4}$	[−1.5, 1.6]	[−2.1, 2.1]	[−1.7, 1.7]	[−2.2, 2.2]	[−8.3, 8.6]	[−12, 12]
$f_{T,6}/\Lambda ^{4}$	[−1.9, 1.9]	[−2.5, 2.6]	[−1.9, 2.0]	[−2.6, 2.6]	[−10, 11]	[−14, 15]
$f_{T,7}/\Lambda ^{4}$	[−4.3, 4.3]	[−5.6, 5.8]	[−4.4, 4.5]	[−5.9, 6.0]	[−20, 20]	[−27, 28]

## Conclusion

The production of $WV \gamma$ events is studied in $e\nu \mu \nu \gamma$, $e\nu jj\gamma$ and $\mu \nu jj\gamma$ final states using 20.2 fb$^{-1}$ of proton–proton collisions at a centre-of-mass energy of $\sqrt{s} = {8\,\text {TeV}}$ recorded with the ATLAS detector at the LHC. The fiducial production cross-section of the $e\nu \mu \nu \gamma$ final state is determined with a significance of 1.4 $\sigma$ (1.6 $\sigma$ expected) and good agreement with the SM prediction at NLO in $\alpha _{\text {S}}$ is observed. Furthermore, upper limits on the production cross-section are derived for the $e\nu \mu \nu \gamma$, $e\nu jj\gamma$, $\mu \nu jj\gamma$ and $\ell \nu jj\gamma$ final states in two fiducial regions: one optimised for the measurement of the process and one optimised for a search for new physics beyond the SM. No deviation from the SM predictions is observed and the results are interpreted in the framework of an effective field theory. Confidence intervals are derived with and without unitarisation for all 14 parameters of anomalous quartic gauge couplings this analysis is sensitive to.

## Footnotes

1 ATLAS uses a right-handed coordinate system with its origin at the nominal interaction point (IP) in the centre of the detector and the *z*-axis along the beam line. The *x*-axis points from the IP to the centre of the LHC ring, and the *y*-axis points upwards. Cylindrical coordinates $(r,\phi )$ are used in the transverse plane, $\phi$ being the azimuthal angle around the *z*-axis. The rapidity (*y*) is defined as $y = \frac{1}{2}\ln \left[ \frac{E+p_{z}}{E-p_{z}}\right]$, where $p_{z}$ is the *z*-component of the momentum and *E* is the energy of the object. The pseudorapidity ($\eta$) is defined in terms of the polar angle $\theta$ as $\eta = -\ln \tan (\theta /2)$. Angular distance is measured in units of $\Delta R \equiv \sqrt{(\Delta \eta )^{2} + (\Delta \phi )^{2}}$.

2 The mass of the $Z$ boson is taken to be $m_{Z} = {91.19\,\text {GeV}}$ [70].
